# A dynamic assembly-induced emissive system for advanced information encryption with time-dependent security

**DOI:** 10.1038/s41467-022-31978-x

**Published:** 2022-07-20

**Authors:** Qian Wang, Biyan Lin, Meng Chen, Chengxi Zhao, He Tian, Da-Hui Qu

**Affiliations:** Key Laboratory for Advanced Materials and Joint International Research Laboratory of Precision Chemistry and Molecular Engineering, Feringa Nobel Prize Scientist Joint Research Center, School of Chemistry and Molecular Engineering, East China University of Science and Technology, 130 Meilong Road, 200237 Shanghai, China

**Keywords:** Self-assembly, Single-molecule fluorescence, Information storage

## Abstract

The development of advanced materials for information encryption with time-dependent features is essential to meet the increasing demand on encryption security. Herein, smart materials with orthogonal and temporal encryption properties are successfully developed based on a dynamic assembly-induced multicolour supramolecular system. Multicolour fluorescence, including blue, orange and even white light emissions, is achieved by controlling the supramolecular assembly of pyrene derivatives by tailoring the solvent composition. By taking advantage of the tuneable fluorescence, dynamically controlled information encryption materials with orthogonal encryption functions, e.g., 3D codes, are successfully developed. Moreover, time-dependent information encryption materials, such as temporal multi-information displays and 4D codes, are also developed by enabling the fluorescence-controllable supramolecular system in the solid phase, showing multiple pieces of information on a time scale, and the correct information can be identified only at a specified time. This work provides an inspiring point for the design of information encryption materials with higher security requirements.

## Abstract (web summary)

The development of advanced materials for information encryption is desirable. Here, the authors report materials that enable orthogonal and temporal encryption of information via dynamic assembly-induced multicolor pyrene-based supramolecular systems.

## Introduction

Currently, storing and delivering information in a secure manner is an essential issue, and many efforts have been devoted to exploring an accessible approach to achieve information encryption^1–5^, such as fluorescent ink^6–9^, holographic anticounterfeiting^10^, and 3D codes^11–19^, where encoded information can be recognized only under specific conditions. Usually, the fabrication of such materials involves embedding a smart molecular system in a polymeric or gel matrix or functional modification of the side chain of the polymers^20–30^, thus endowing the materials with stimuli-responsive and dynamic properties^31–33^. Developing dynamic molecular systems is therefore important for fabricating advanced data encryption materials.

A smart fluorescence system is indeed a potential candidate for fabricating information encryption materials due to its security in natural light^34^, i.e., information can be recognized only under UV light. In addition, the fluorescence (either intensity or wavelength) can be controlled by an external stimulus^35–48^, providing an accessible approach to control information display. By taking advantage of these features, Zhao and coworkers recently developed a light-controlled information encryption material by using a photoresponsive supramolecular coordination polyelectrolyte as a fluorescence ink^34^. A multifluorescent supramolecular hydrogel system to fabricate 3D/4D codes was introduced as an applicable approach to encrypt information, as well demonstrated by Sessler and Tang et al.^49–51^. Moreover, information self-destructing materials^52–54^, exhibiting a higher level of security, were developed by Chen and coworkers by introducing a hydrolysis reaction into a fluorescent hydrogel^55^. These contributions provided inspiring insights for the design and fabrication of materials for information encryption. Nevertheless, the fabrication of such materials, especially those that exhibit advanced encryption properties, such as orthogonal encryption, selective encryption or even time-dependent encryption, remains challenging.

**Fig. 1:**
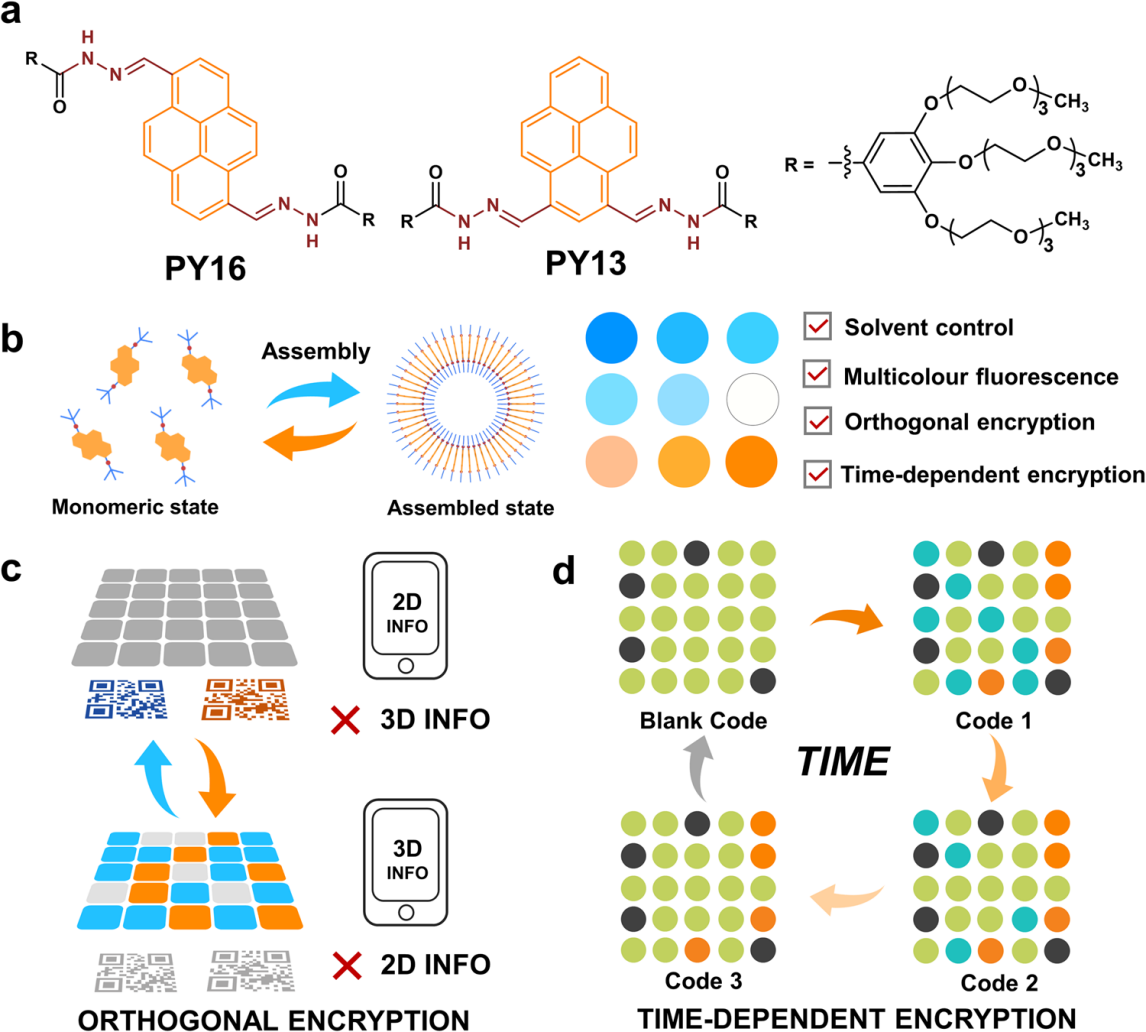
The construction of a dynamic assembly-induced emissive system and advanced information encryption material with time-dependent security. **a** Molecular structures of two compounds. **b** Dynamic self-assembly and resultant multicolour fluorescence. **c** 3D code and **d** 4D code for information encryption.

Here, we generate an information encryption material based on a pyrene-based multicolour fluorescence supramolecular system with time-dependent dynamic fluorescent signals (Fig. 1). As the pyrene fluorophore features a strong redshifted excimer emission, the molecular fluorescence could be regulated from the blue to orange–red region, including white-light fluorescence, by controlling supramolecular self-assembly through solvent composition tailoring. A material for information encryption with a switchable fluorescent colour 3D code function is subsequently designed and fabricated. Information stored in the coloured code can be accessibly read using a smartphone under UV light, but not in natural light, and is able to transform through physical action or solvent control. Moreover, by incorporating 2D codes into 3D code, a more advanced approach to encrypt data, orthogonal encryption is achieved, i.e., 2D code information could be read in natural light while 3D code is invisible, and the converse result is obtained under UV light. Such encryption is controllable. The integration of 3D code not only transforms the 3D code information but also exposes the 2D code. Both 2D and 3D code information can thus be read under UV light. Furthermore, for materials that encrypt information on a time scale, such as time-dependent multi-information displays, 4D codes are successfully developed by enabling the smart fluorescence system in the solid phase. Solvent treatment results in dynamic, transient and reversible changes in the fluorescence colour, which would autonomously revert back to the initial colour over time. This time-gated fluorescence change enables the information to be dynamic on a time scale, and therefore, a series of information is obtained with time-dependent features. The correct information can be recognized at only a specified time and would self-erase after being read. Taking advantage of this time-dependent security, the complexity of the current encryption materials is significantly increased, paving the way towards sophisticated implications with higher security requirements. In this work, smart materials with orthogonal and temporal encryption properties are successfully developed based on a dynamic assembly-induced multicolour supramolecular system.

## Results

### Molecular design

The fabrication of the multicolour fluorescence supramolecular system involved the control of supramolecular assembly of pyrene-based amphiphilic molecules. In a previous study, we demonstrated that pyrene fluorophore modification with an acylhydrazone unit resulted in a broadened fluorescent spectrum window, enabling the fluorescence emission to cover from the blue to green–yellow region^52^. To further redshift the fluorescence emission and cover a wider range of the fluorescence spectra, compound **PY16**, a pyrene unit decorated with two acylhydrazone units at the 1- and 6-positions, was prepared by the condensation of a hydrazide and 1,6-diformylpyrene catalyzed by trifluoroacetic acid (Supplementary Fig. 1). The compound terminus was modified with six oligoethylene glycol chains to i) tune the amphiphilicity of the resulting pyrene derivative to facilitate its assembly in water monitored by spectroscopic and microscopic techniques and ii) provide good solubility in both organic solvents and water so that we could control the molecular assembly of the pyrene-based system by the designed solvent media. In addition, to exploit whether the substituted position would influence the molecular fluorescence, the reference compound **PY13**, with two acylhydrazone units at the 1- and 3-positions, was also synthesized following the same process. The molecular structures of the two target compounds were confirmed by NMR and mass spectrometry (Supplementary Figs. 2–7).

### Assembly-induced fluorescence

**Fig. 2:**
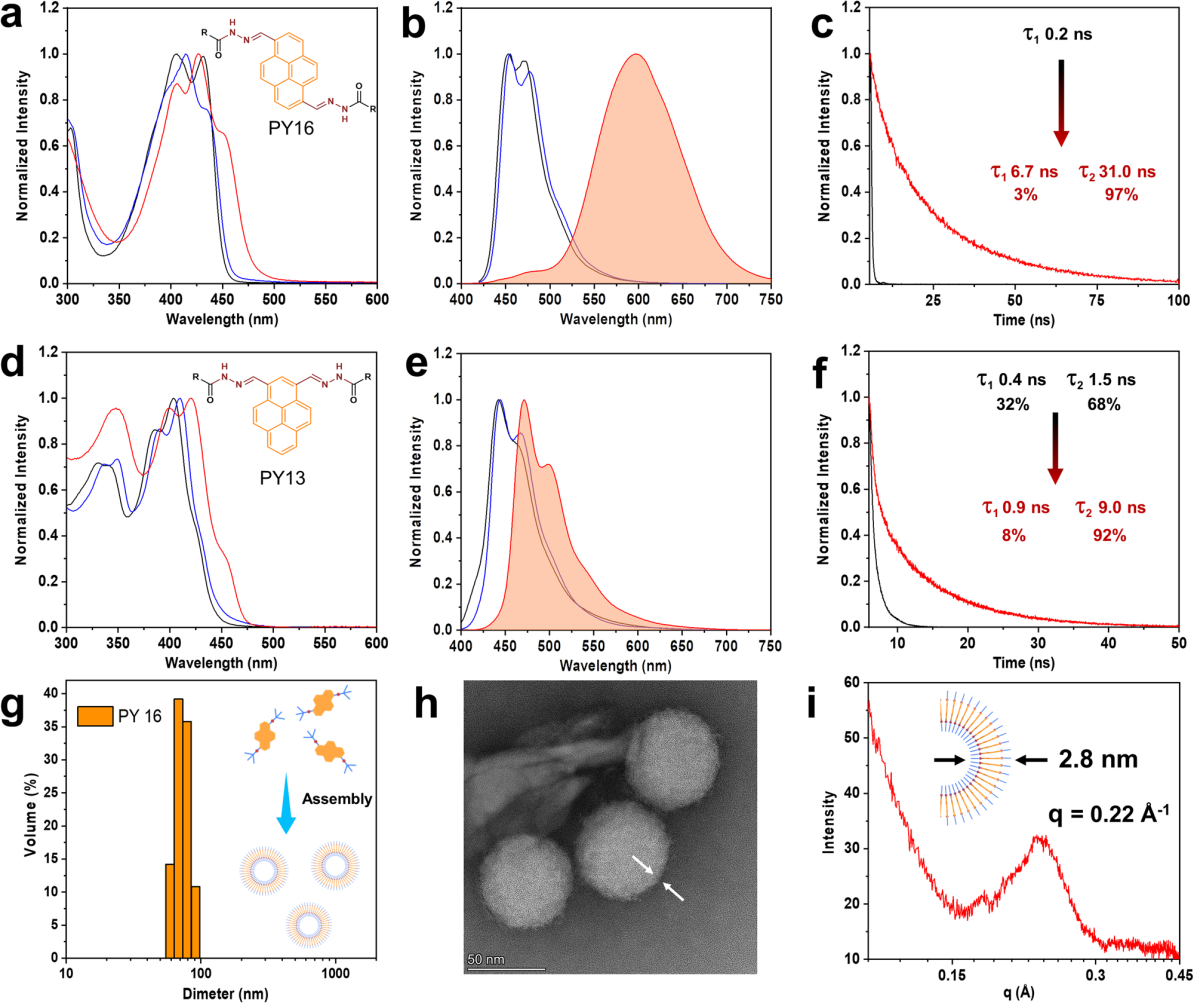
Amphiphilic self-assembly and photophysical studies of two compounds (25 °C, 20 μM). **a**, **d** UV–Vis absorption spectra and **b**, **e** fluorescence spectra of the compounds in various solvents (black line: dichloromethane, blue line: dimethyl sulfoxide, red line: water), λ_ex_ = 380 nm. **c**, **f** Corresponding time-resolved fluorescence decay (fitting) curves of the compound (black line: dimethyl sulfoxide, red line: water), λ_ex_ = 380 nm. **g** DLS data of **PY16** in an aqueous solution (25 °C, 0.1 mM). **h** TEM image of **PY16**, which self-assembles to form vesicles in an aqueous solution (25 °C, 0.1 mM). **i** Synchrotron radiation SAXS pattern of the vesicles.

To investigate the solvent effect on the supramolecular self-assembly process, UV–Vis absorption and fluorescence measurements were carried out. For compound **PY16**, absorption peaks with vibronic features at 395 nm and 430 nm were observed in dichloromethane (DCM), suggesting the nonassembled status of the compound (Fig. 2a). This status was also confirmed by the appearance of the typical monomeric emission peaks in the blue region (450 nm and 480 nm). In addition, similar absorptions and fluorescence peaks with slight redshifts were observed in a more polar solvent (dimethyl sulfoxide) than in dichloromethane. This phenomenon suggested that **PY16** resides in the monomeric state in both organic solvents, implying that solvent polarity has a small influence on optical molecular properties (wavelength). In contrast, compound **PY16** showed a broad absorption peak with a considerable redshift in aqueous solutions, suggesting the stacking of the pyrene units, i.e., the occurrence of the assembly. Such assembly was further confirmed by the changes of the fluorescence properties (Supplementary Fig. 8): i) the appearance of an emission peak at 600 nm, with the strong long-wavelength, broad and unstructured characteristics (Fig. 2b); ii) the observation of a much longer, biexponential decay (3% arising from the short-lived component of 6.7 ns and 97% arising from a long-lived component of 31 ns) in aqueous solution compared to the short lifetime (τ_s_) of 0.2 ns for monomers in the dimethyl sulfoxide (DMSO) solution (Fig. 2c); and iii) the increase in the quantum yield (from 8.0% to 39.5%, Supplementary Fig. 9). Although **PY16** has been assembled in this concentration, the emission at 450 nm and 480 nm with low intensity (~0.1 a.u) was still visible, suggesting the presence of monomers.

To better understand the assembly behaviour of compound **PY16**, concentration-dependent (4.5 × 10^−7^−5.5 × 10^−6 ^M) fluorescence spectra of compound **PY16** in aqueous solution were obtained, showing a gradual enhancement of the excimer emission at approximately 600 nm (Supplementary Fig. 10). Notably, a distinctive slope transition point in the linear function of the fluorescence intensity at 600 nm versus concentration revealed a critical assembly concentration (CAC) of 3 μM. In 5.5 μM aqueous solution, the low-intensity monomer emission at approximately 450 nm (~0.2 a.u.) was still visible. To further investigate the relationship between monomer and excimer emission, the fluorescence spectra of **PY16** aqueous solutions with higher concentrations from 4.5 × 10^−6 ^M to 8.9 × 10^−5 ^M were obtained. Here, excimer emission was dramatically increased above the CAC. As a result, the monomer emission was invisible in a high concentration solution (8.9 × 10^−5 ^M). In addition, dynamic light scattering (DLS) measurements were carried out to examine the assembled structural particle size, showing a relatively narrow distribution with a diameter of approximately 80 nm (Fig. 2g). Transmission electron microscopy (TEM) further revealed the assembled morphologies (Fig. 2h, Supplementary Fig. 11), vesicles, with a diameter corresponding to that from the DLS measurements. Moreover, the structural detail, vesicle wall thickness, was confirmed according to the Bragg equation (d = 2π q^−1^) determined by the small-angle X-ray scattering (SAXS) experiment, giving a value of approximately 2.8 nm (Fig. 2i)^56^.

In contrast, compound **PY13** showed a sharp emission peak with vibronic features in both organic and aqueous solvents, suggesting that the assembly of **PY13** did not occur in aqueous solution (Fig. 2e). Such behaviour was further confirmed by concentration-dependent (0.5–100 μM) fluorescence spectra (Supplementary Fig. 12): i) the observation of a sharp emission peak in both dilute (0.5 μM) and concentrated solutions (100 μM) instead of a broad peak and ii) the linear changes in the fluorescence intensity at 470 nm. Although slight redshifts in the absorbance, fluorescence, and increase of the fluorescent lifetime (8% arising from the short-lived component of 0.9 ns and 92% arising from a long-lived component of 9.0 ns) as well as the quantum yield (from 6.7% to 13.2%) were observed (Fig. 2d–f, Supplementary Fig. 13), these may be attributed to solvent polarity changes^57^. The absolute solid fluorescence quantum yield of compounds **PY16** and **PY13** was also performed, giving a value of 15.4% and 6.6% (Supplementary Fig. 14), respectively. These optical findings suggested that the chemical design regarding the substitution position had a significant impact on amphiphilic self-assembly and optical properties.

**Fig. 3:**
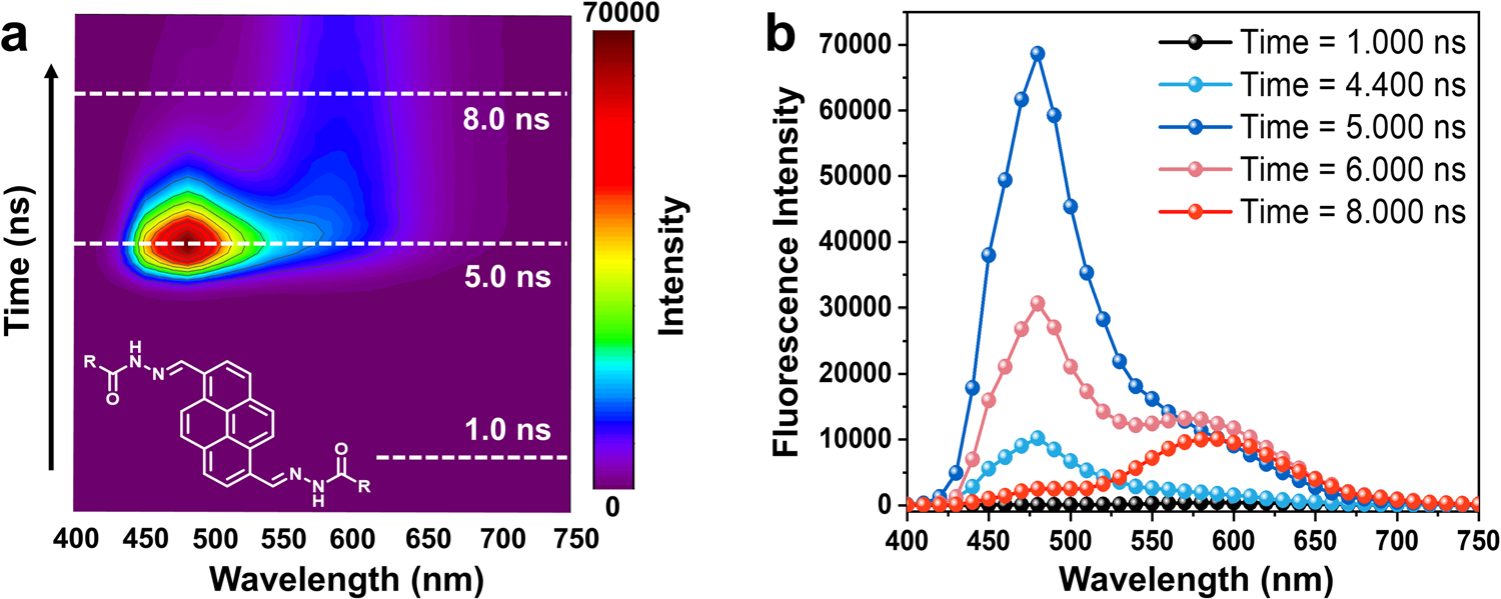
Excimer formation process. Time-resolved fluorescence (**a**) mapping and (**b**) spectroscopy of compound **PY16** in aqueous solution (25 °C, 20 μM). The solution of **PY16** contained 10% (volume ratio) dimethyl sulfoxide to enhance monomeric emission. The redshift of the emission over time indicated the formation of excimers.

The strong long-wavelength emission induced by assembly prompted us to explore the formation process. Considering the redshift and broad and unstructured characteristics of the emission peak, we hypothesized that the compounds formed excimers in their assembled states upon excitation by UV light. To confirm this, the excitation spectra were acquired (Supplementary Fig. 15). Excitation of the DMSO solution (20 μM, monomer) resulted in emission similar to that in the aqueous solution (20 μM, assembly), suggesting the presence of the excimer. Furthermore, to gain in-depth insight into the excimer formation process, time-resolved emission measurements were carried out (Fig. 3, Supplementary Fig. 16). A blue emission peak was obtained at approximately 480 nm (0–5.0 ns), which was similar to the monomeric emission in organic solvents. As time elapsed (5.0-8.0 ns), this emission peak gradually decreased, which was accompanied the appearance of a long wavelength emission band (600 nm). Such a time-dependent change clearly revealed the formation process of the excimers.

**Fig. 4:**
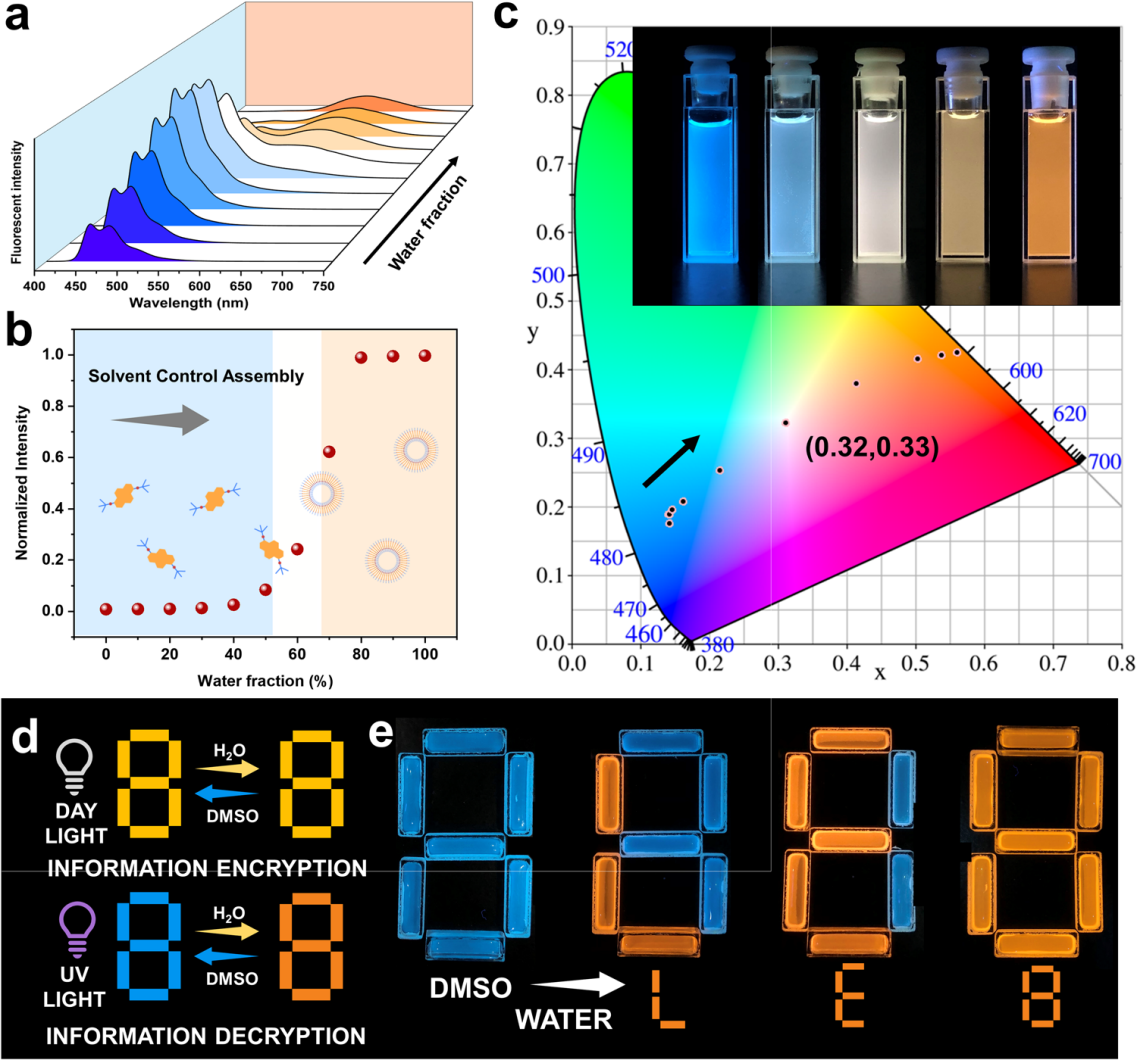
A multicolour fluorescence system controlled by tailoring the solvent composition. **a** The optical features of the emissive compound **PY16** monitored by fluorescence spectroscopy upon increasing the water content (from blue to orange), λ_ex_ = 380 nm. **b** Recorded fluorescence intensity changes in the excimer emission peak (600 nm) with different water fractions. **c** CIE 1931 chromaticity diagram of compound **PY16** with a steadily increasing water fraction. Inset: fluorescence images of **PY16** in DMSO/H_2_O mixtures with different water fractions under UV light exposure, λ_ex_ = 380 nm. **d** A schematic illustration of the information encryption-decryption process. **e** Fluorescence images of multiple pieces of information made from **PY16** solution and written by adding water.

At this point, it is possible to conclude that compound **PY16** self-assembled into vesicles with strong redshifted excimer emission in aqueous solution, whereas it exhibited blue fluorescence in organic solvents. In other words, molecular assemblies with distinct optical and morphological properties were highly dependent on the solvent media, which offered an inspiring approach to achieve control over the optical information outcome carried by these assemblies by varying the solvent composition. The fluorescence spectra of **PY16** in DMSO/H_2_O mixed solutions revealed a transition from monomeric to assembled species upon increasing the water fraction, which was accompanied by decreases in the blue emissions at 450 nm and 480 nm and an increase in the excimer emission at 600 nm (Fig. 4a). These fluorescence changes were demonstrated by the corresponding plot of excimer fluorescence intensity versus the water fraction (Fig. 4b). Upon increasing the water content in the aqueous DMSO mixtures, the fluorescence varied from blue (0.16, 0.18) to orange (0.56, 0.42), including a white light emission (at 0.32, 0.33) (Fig. 4b, c). This change was also recorded by the fluorescence image of the compound solution (inset in Fig. 4c). Such solvent-dependent fluorescence properties enabled these compounds to be potential candidates for message encryption and storage materials^23,24^. We used a quartz container as a template to develop such encryption material (Fig. 4d, e). **PY16** in pure DMSO was filled in the quartz to make an “8” pattern, which appeared as yellow in daylight and fluorescent blue under UV light. The addition of water resulted in the assembly of the compound and therefore exhibited orange fluorescence, showing a strong contrast to the solutions without water treatment under UV light. In this way, massages were written and stored. Such massages were secured because they could be visualized only under UV light, while no obvious changes could be observed under daylight (Supplementary Fig. 17). By controlling the addition of water, massages ‘L’, ‘E’, and ‘8’ can therefore be easily written.

### Dynamic 3D code

**Fig. 5:**
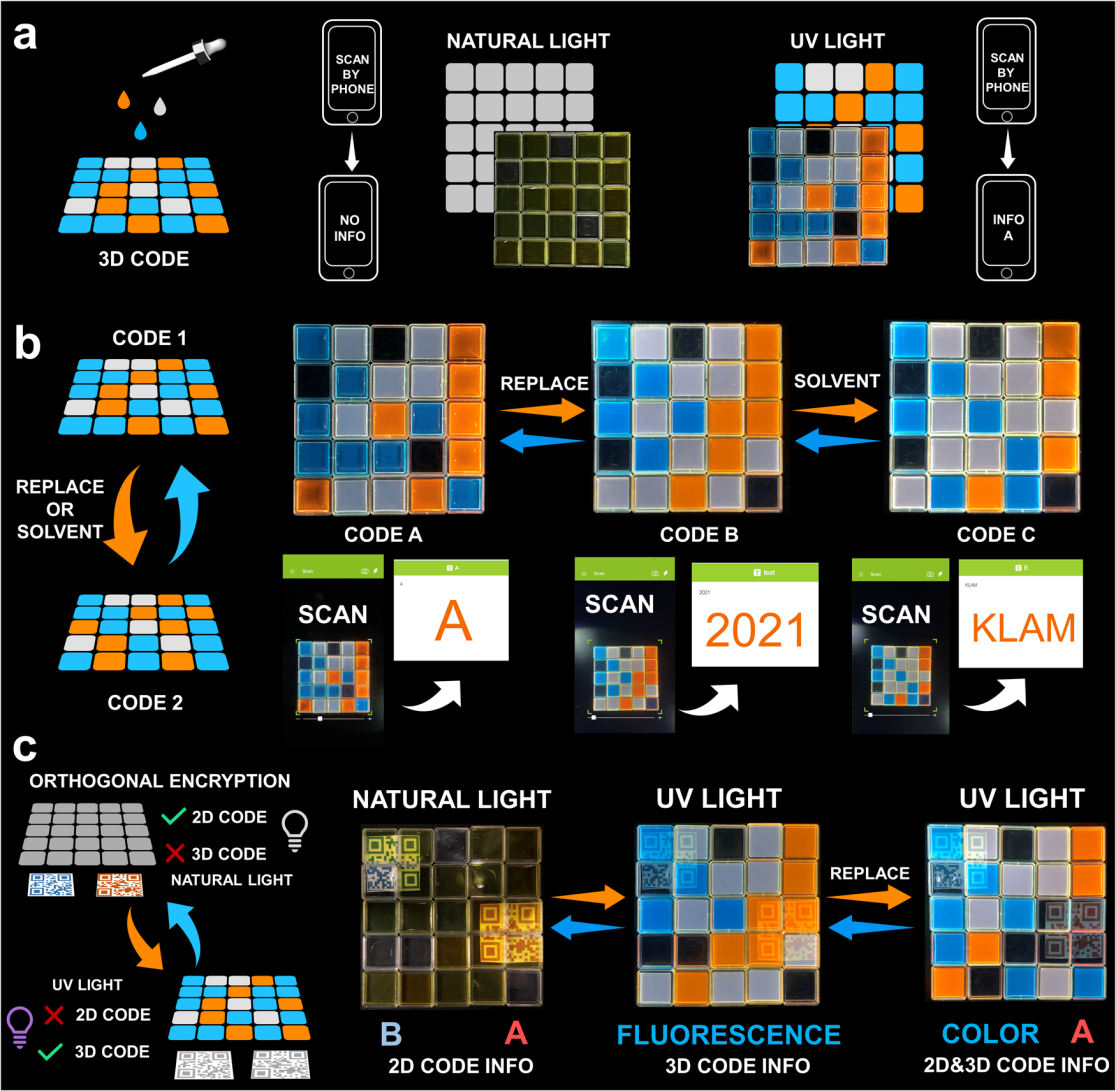
Dynamic 3D code for information encryption. **a** Schematic representations of the 3D colour code formation by adding **PY16** solution dropwise to quartz containers. Blue, white and orange represent the DMSO, DMSO/H_2_O, and H_2_O solutions, respectively. The daylight and fluorescence images of the 3D code are included. The encoded information could be recognized under UV light but could not be identified under natural light. **b** Schematic representations for the 3D code transformation by means of physical action or introducing solvent. Photographs of the 3D codes under UV light are presented, and the corresponding information was read using a smartphone. **c** A visual scheme illustrating the information orthogonal encryption process and photographs of new codes combining 2D code and 3D code.

Recently, 3D colour codes, composed of three colours (usually but not limited to blue, green and red), have attracted both scientific and industrial interest due to their higher information storage capacity and better security compared to conventional codes^11–16^. However, the development of 3D colour codes, especially those that can be transformed, is still rare. This dynamic and controllable multicolour fluorescent system could potentially be a candidate for developing advanced 3D colour codes. This 3D colour code could be constructed by arranging boxes loaded with **PY16** solution in a specific way on a black substrate (Fig. 5a). The stability of compound **PY16** was confirmed by NMR spectroscopy (Supplementary Fig. 18). By controlling the solvent composition, three fluorescent colours, blue, white and orange, could be achieved by three different solvent environments, DMSO, DMSO/H_2_O (4:6) and water, respectively. The encoded information was secure because the three colours could be visualized only if UV light was applied. In this situation, the 3D code was recognized by smartphone software app named COLORCODE, and information was interpreted, translated and stored using COLORCODE (Supplementary Movie 1). In natural light, the boxes displayed similar colours, and therefore, the information was invisible. Moreover, the encoded information can be transformed by physically replacing the boxes (Fig. 5b). For example, the change in the number and sequence arrangement of the blue-, orange- and white-coloured boxes resulted in the reversible switching of patterns between code A and code B. Correspondingly, the information code pattern interpreted as “A” could dynamically transform into that of “2021” (Supplementary Movie 2). Such transformation can also be achieved by solvent control, i.e., for each of the boxes, upon adding water into the DMSO solution, the compound (self-)assembled, giving rise to a change in fluorescent colour (from blue to white or orange). This change resulted in the appearance of code C, and the corresponding encoded information “KLAM” was read under UV light (Supplementary Movie 3). Therefore, 3D fluorescent colour codes, a kind of dynamic information storage, encryption and transformation material, were successfully developed. Compared to other fluorescent 3D codes reported, the construction strategy is easy to conduct, and the resulting codes can be transformed in a simple manner.

We therefore hypothesize that we can develop codes with a higher level of security by using an orthogonal encryption strategy, which allows the data to be selectively read. This suggested that a single piece of material (code) carries multiple pieces of information, while only one piece of information can be read at a time. To do so, 2D code was incorporated into 3D code, i.e., two 2D codes were printed on paper and placed between the boxes and black substrate, forming a sandwich-like structure (Fig. 5c and Supplementary Fig. 19). In natural light, the solution in the box was transplant, and therefore, the 2D codes could be recognized by software, identified as the encoded information “A” (red code) and “B” (blue code) (Supplemnetary Movie 4 and 5). However, such information could not be identified under UV light because the fluorescent colour pattern covered the 2D code, while the 3D code could be recognized, and the information “fluorescence” stored in the 3D code was read (Supplementary Movie 6). In other words, UV light allowed us to simultaneously identify information carried by the 3D code, where the information in the 2D codes was successfully hidden from being interpreted, while the opposite result was obtained when exposed to natural light. Therefore, orthogonal encrypt information was achieved. This strategy was different from the code in code approach that exhibits good information storage capacity, where all information was decrypted under UV light, as reported by Ji and coworkers^51^. Our strategy focused on data security integration. Notably, orthogonal encryption is controllable. Physical movement of the allocation of different coloured boxes resulted in the overall change of the fluorescent pattern, which was in response to the construction of a new 3D code and the selective encryption of the 2D code. In this situation, both 3D codes and the decrypted 2D code were recognized under UV light. For example, when the orange and white boxes covering the red 2D code were replaced with four empty transparent boxes, the red 2D code was decrypted while the blue 2D code was still encrypted, leading to the identification of the new 3D code’s information “color” and red 2D code’s information “A” (Supplementary Movie 7–8). This controllable orthogonal encryption strategy allows the information to be selectively identified, which is of particular interest for designing and fabricating information encryption material with high-level security.

### Time-dependent information encryption

**Fig. 6:**
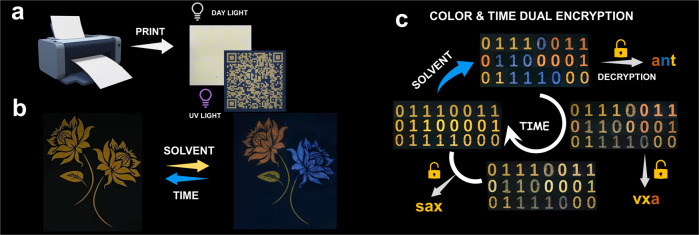
Security features of fluorescent ink. **a** Schematic representations for the printing experiment and a photograph of the printing pattern 2D code decrypted under UV light. **b** Fluorescence images of the printing pattern before and after treatment with organic solvent (blue) and water (orange). The fluorescence change was reversible and transient, reverting back to yellow with time. **c** Fluorescent photographs of the printing binary code, illustrating colour and time dual encryption functions.

Solid-phase information encryption is more efficient than that of the liquid phase, addressing the question of whether we could develop solid-phase information encryption materials. To achieve this, a print test was carried out. Although a pale-yellow colour was observed in natural light, the printing pattern was not clear enough to be recognizable. In contrast, when the paper was exposed to UV light, a much brighter yellow fluorescent pattern, the 2D code, was observed (Fig. 6a). This suggests that the encryption material was endowed with information security under natural light/visible light.

The solvent-controlled fluorescence has guided us to explore the possibility that the fluorescent colour can be tuned on paper, endowing the information encryption to be programmable. More specifically, a rose pattern (Fig. 6b left) was treated with water, which resulted in a fluorescence colour change from yellow to orange. This may be attributed to the excimer emission induced by the assembly of compound **PY16**. Conversely, the addition of the organic solvent N-propanol gave rise to monomeric fluorescence, and therefore, the rose pattern (Fig. 6b right) exhibited a blue fluorescent colour. We experimentally observed a dynamic, transient fluorescence colour that was changeable on a time scale, owing to solvent evaporation, i.e., both blue and orange fluorescence would revert back to yellow with time. The duration of the blue and orange fluorescence display can be regarded as the lifetime, which is affected by the rate of solvent evaporation. Solvents with a relatively slower evaporation rate, such as water, tended to have a longer lifetime (4 mins), while a short lifetime (2 mins) was observed when using N-propanol (Supplementary Movie 9). This time-gated fluorescence with different colours has led to the following hypothesis: it is possible to develop an advanced information encryption material with two encryption approaches, colour and time. The correct information can be recognized by only distinguishing different colours at a specific time. To do this, the binary code was printed on the paper, and the corresponding information “sax” was identified under UV light (Fig. 6c). The addition of water and N-propanol resulted in three different colour codes that were used as keys to decrypt information. By classifying and arranging the same colour code in order, information “a”, “n” and “t” were recognized as the orange, blue and yellow codes, respectively. In this way, the correct information “ant” was then identified. This piece of information was dynamic on a time scale. The blue-colour numerical codes self-erased over time, while the orange codes remained unchanged due to the different evaporation rates of the solvents, leading to the self-erasing effect as well as the disappearance of encoded information “n”. Recognizing the binary code with yellow and orange colours gave rise to the false information “vxa”. Subsequently, the orange code could also be self-erased, leading to the reversion of the information (from “vxa” to “sax”). These two encryption procedures, especially time-dependent encryption that displayed multiple pieces of information on a time scale, endow the material with advanced information protection capabilities compared to switchable (on/off) information encryption.

**Fig. 7:**
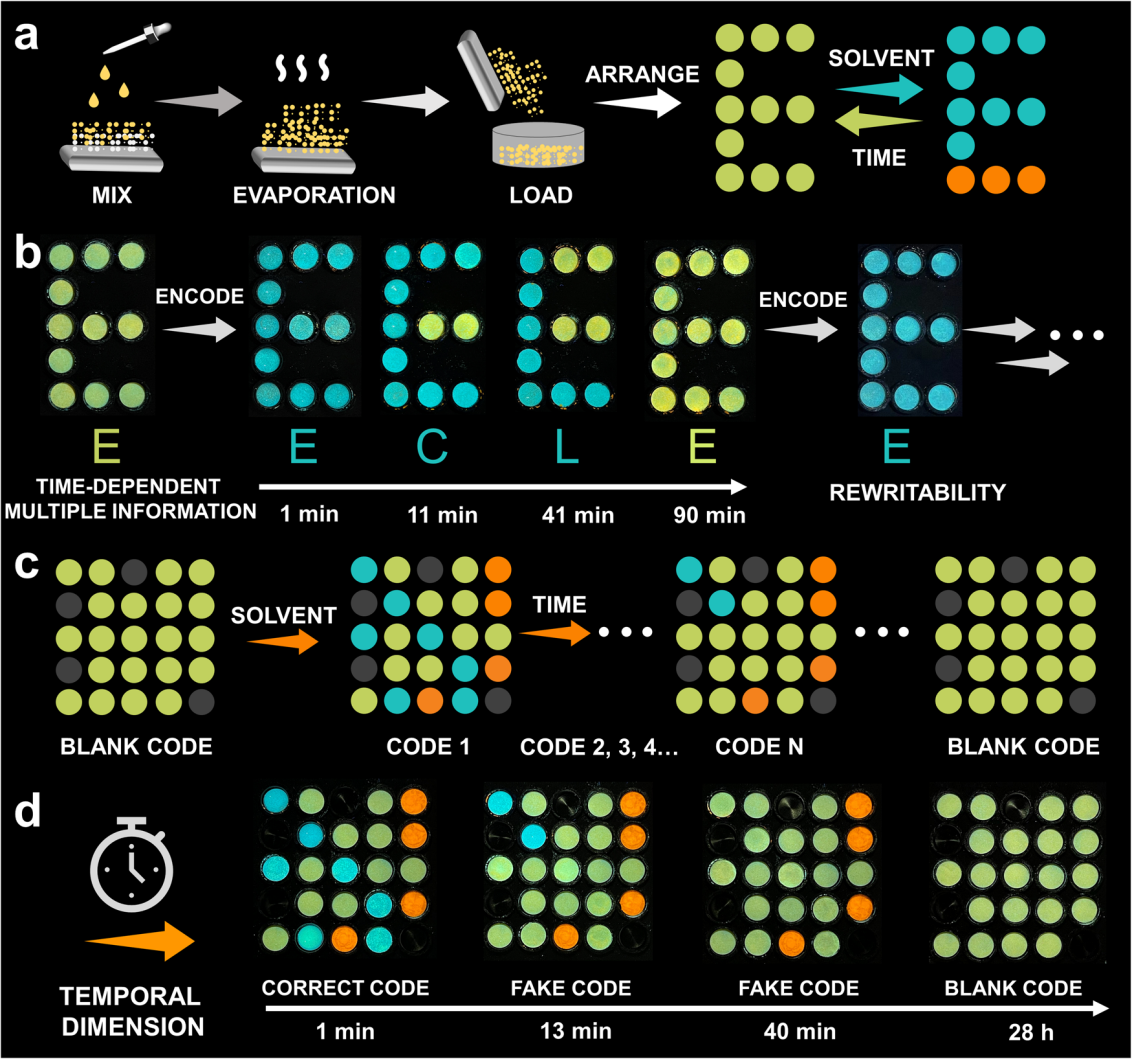
A visual demonstration of the information encryption material on a time scale. **a** Schematic representations of the process of fabricating such a material and its dynamic fluorescence. Yellow dots represent the dry silica gel powder loaded with compound **PY16**, which turned blue and orange after adding dichloromethane and water, respectively. **b** Photographs of the encrypted letter “E” before and after treatment with dichloromethane, displaying time-dependent multiple pieces of information in which the correct piece can be identified only at a specified time. **c** Schematic representations and fluorescence images (**d**) of the dynamic 3D code on a time scale. The blue- and orange-coloured information self-erased into yellow as time passed, leading to the generation of a 4D code with time-dependent security.

Nevertheless, such reversibility is not ideal because the dye loaded on the paper would dissolve when the solvent is applied, hindering its use in practical applications (Supplementary Fig. 20). The development of a time-dependent material with good reversibility is therefore necessary. To overcome this limitation, **PY16** was loaded on silica gel, which has a strong adsorption capacity for organic compounds. Silica gel (2.7 g) was mixed with 24 ml of compound DCM solution (3 mM) and then evaporated. The resulting powder was loaded in a black container and exhibited yellow fluorescence (Fig. 7a). The addition of the organic solvent resulted in blue fluorescence with high quantum yield (from 23.4% to 65.4%), which was in strong contrast to its initial emission (Supplementary Fig. 21). Therefore, information could be written by adding organic solvent followed by visualization under UV light (blue fluorescence). Such information was transient and dynamic on a time scale, owing to solvent evaporation. When the remaining solvent was totally evaporated, the fluorescence reverted to yellow, and therefore, the information was erased (Supplementary Movie 10). Additionally, the lifetime of the information maintaining readability can be controlled by the amount of each solvent contained, e.g., the higher the solvent content was, the longer the lifetime was.

By taking advantage of this feature, we added different amounts of DCM to the container, enabling the materials to have different lifetimes. Different pieces of information in a sequence of ‘E’, ‘C’, ‘L’ would then be read following a time course of 1 min, 11 min, 41 min, 90 min (Fig. 7b). This implied that the material exhibited a time-dependent information encryption characteristic, i.e., it was accessible to identify only information “C” when the solvent evaporation time reached 11 minutes; once the evaporation time became longer or shorter, false information “E” or “L” or blank information (yellow colour “E”) was then identified. Other information, such as “T” and “8”, was also encoded in this material, showing various pieces of information on a time scale (Supplementary Fig. 22). Moreover, this material exhibited good rewritability: information can be rewritten by adding DCM again, and the writing-erasing process can be repeated over ten times without damaging the materials (Supplementary Figs. 23 and 24). It is worth noting that self-erasing materials also exhibit time-dependent information encryption characteristics. The information they carry usually involves two states: displayed and erased. Multiple information presented on a time scale has rarely been reported, owing to the complex nature of this data display process. However, this function can be easily achieved in this material by achieving assembly-induced dynamic fluorescence by controlling the solvent content, which allows it to be superior in terms of time-dependent data security.

This time-dependent security led us to propose a conceptual design: fabricating a dynamic 3D code on a time scale, i.e., 4D code, in a simple and controllable manner (Fig. 7c, Supplementary Fig. 22). To achieve this goal, silica gel containing compound **PY16** was loaded in a black container and then arranged to a blank code without any encoded information. Solvent introduction led to the fluorescent colour change, and therefore, four distinct colours, black (container colour), blue, yellow and orange, were observed under UV light. A 3D code (code C) with the stored information “KLAM” was then constructed (Fig. 7d). Such 3D code was dynamic on the time scale because the blue colour would revert back to yellow over time, which was concomitant with solvent evaporation. Over time, a series of codes were obtained after the solvent had evaporated for 1 min, 13 min, 40 min, and 28 h accordingly. As a result, a 3D code system was transformed into a 4D code system with time-dependent information encryption functions. Notably, information was accessible at only a specified time (“KLAM” at 1 min) and recognition was inhibited during the rest of the time course. Once the solvent was completely evaporated, a blank code with yellow fluorescence was obtained, and a new code could be constructed by introducing the solvent again, endowing the code with good reusability.

## Discussion

In summary, an advanced information encryption material that encrypted information in an orthogonal and temporal manner has been successfully developed by taking advantage of a dynamic assembly-induced emissive supramolecular system. This system was composed of amphiphilic pyrene dyes bearing hydrophilic triethylene glycol chains for decent solubility in both water and organic solvents, providing an accessible approach to control molecular assembly by tailoring the solvent composition. As a result, tuneable fluorescent emissions ranging from blue to orange, including pure white-light emission, were achieved. Relying on this controllable multicolour fluorescence character, an information encryption material, 3D code, was then developed. The information stored in this 3D code can be recognized by smartphones under UV light but is invisible in natural light, and it can be transformed through physical actions or solvent control. Additionally, orthogonal and selective information encryption was achieved by incorporating 2D code into 3D code. This encryption method allowed the information (2D, 3D code or both) to be selectively read, exhibiting promising potential for multiple information encryption. Moreover, advanced information encryption materials, such as time-dependent multiple information displays and 4D codes, were developed by enabling the dynamic assembly-induced emissive system in the solid phase, showing multiple pieces of information on a time scale, and the correct information can be identified at only a specified time. We expect these findings to serve as an inspiring starting point for the fabrication of advanced information encryption materials in a temporal manner by using a supramolecular emissive system with controllable fluorescence.

## Methods

### Materials

Chemicals were purchased from TCI, Adamas-beta® and Sigma-Aldrich and used without any further purification. All solvents were reagent grade and were dried and distilled prior to use according to standard procedures.

### Compound synthesis and purifification

The synthetic details of compounds **PY16** and **PY13** can be found in Supplementary Fig. 1. The molecular structures are determined using ^1^H NMR, ^13^C NMR spectroscopies, and high-resolution electronic spray ionization (ESI) mass spectrometry.

### Equipment

Chemicals were weighed on analytical balances METTLER-TOLEDO, ME204T/02. Flash column chromatography was performed using silica gel (Greagent, 200-300 mesh) to purified crude products. The ^1^H and ^13^C NMR spectra were measured on a Brüker AV-400 and AV-600 spectrometer at 298 K. The ESI-MS was experimented on a LCT Premier XE mass spectrometer. The UV/Vis absorption spectra data were documented by a Shimadzu UV-2600 UV-Vis spectrophotometer and the fluorescence spectra were acquired by a Shimadzu RF6000 spectrofluorophotometer. Transmission electron microscopy (TEM) characterization was performed using ThermoFisher Talos F200X (FETEM, 200 kV). DLS was measured on MALVERN, ZETA SIZER, ModelZEN3600 at 298 K.

### Ink printing tests

Printing tests were performed on a Canon inkjet printer (MG 2400) and Canon PG-845 FINE cartridge, using paper without optical brightener. Ink in black cartridge was replaced by the compound PY 16 ethanol solution (1 mM). Fluorescence images that printed on the paper was visible under UV light. The QR code was scanned by a commercially available smartphone APP. The time-dependent information encryption was performed by spraying the solvent to the paper with a mask in the incubator (298 K). The pictures and movies were recorded under UV light.

### Preparation of 3D code

Small quartz boxes (12 × 12 × 4 mm) were placed on a 20 × 20 cm black paper. The DMSO solutions of compound **PY16** with different water contents (0%, 60% and 100%) were added by using pipette. These boxes exhibited blue (0%), white (60%) and orange (100%) fluorescence colour under UV light. The empty boxes did not contain any solution and therefore exhibited black colour. 3D code was consisted by arranging these four colour boxes in a specific order and scanned by a commercially available smartphone APP. The addition of the solvents (DMSO or water) or physical action resulted in the change of the colour order and therefore a new 3D code was generated. Pictures were recorded under natural with/without UV light.

### Preparation of 4D code

The silica gel containing compound **PY16** was loaded on the small black containers (radius: 5 mm) and then arranged in a specific order on a black cardboard (20 × 20 cm) to fabricate a blank code. The blank code was putted into an incubator (298 K) before different amount of solvents (DCM or water) was adding. The code showed time-dependent colour change owing to the evaporation of the solvent and such change process was recorded in Supplementary Movie 10. Pictures and movies were recorded under UV light.

## Supplementary information

Supplementary Information

Description of Additional Supplementary Files

Supplementary Movie 1

Supplementary Movie 2

Supplementary Movie 3

Supplementary Movie 4

Supplementary Movie 5

Supplementary Movie 6

Supplementary Movie 7

Supplementary Movie 8

Supplementary Movie 9

Supplementary Movie 10

## Data Availability

All data are available within the article and its Supplementary Information. Additional data are available from the corresponding authors upon request.

## Code availability

The 3D code and corresponding scan software used in the present study is COLORCODE® (http://colorzip.com). At the time of publication this information reading application was available free of charge for use in code scanning, but not to the authors’ knowledge for any other purpose.
