# Supplementary material for: A dynamic assembly-induced emissive system for advanced information encryption with time-dependent security
Source: Nat Commun. 2022 Jul 20;13:4185. doi: 10.1038/s41467-022-31978-x (PMC9300691; doi:10.1038/s41467-022-31978-x)
Supplement: Supplementary file 1 — Supplementary Information [file 41467_2022_31978_MOESM1_ESM.pdf]

# Supplementary Information

# A Dynamic Assembly-Induced Emissive System for Advanced Information Encryption with Time-Dependent Security

Qian Wang[1], Biyan Lin[1], Meng Chen[1], Chengxi Zhao[1], He Tian[1], and Da-Hui Qu[1]*

[1]Key Laboratory for Advanced Materials and Joint International Research Laboratory of Precision Chemistry and Molecular Engineering, Feringa Nobel Prize Scientist Joint Research Center, School of Chemistry and Molecular Engineering, East China University of Science and Technology, 130 Meilong Road, 200237 Shanghai, China.

*Corresponding author. Email: dahui_qu@ecust.edu.cn

# Table of Contents

1. **Supplementary Methods……............................................................................. 3-8**

   **1.1 Synthesis and characterization of compounds…....…............................ 3-8**

2. **Supplementary Figures……........................................................................... 9-16**

   **2.1 Amphiphilic self-assembly......................…........................................ 9-12**

   **2.2 Information encryption materials……................................................... 13-16**

3. **Supplementary References................................................................................... 17**

# 1. Supplementary Methods

## 1.1 Synthesis and characterization of compounds

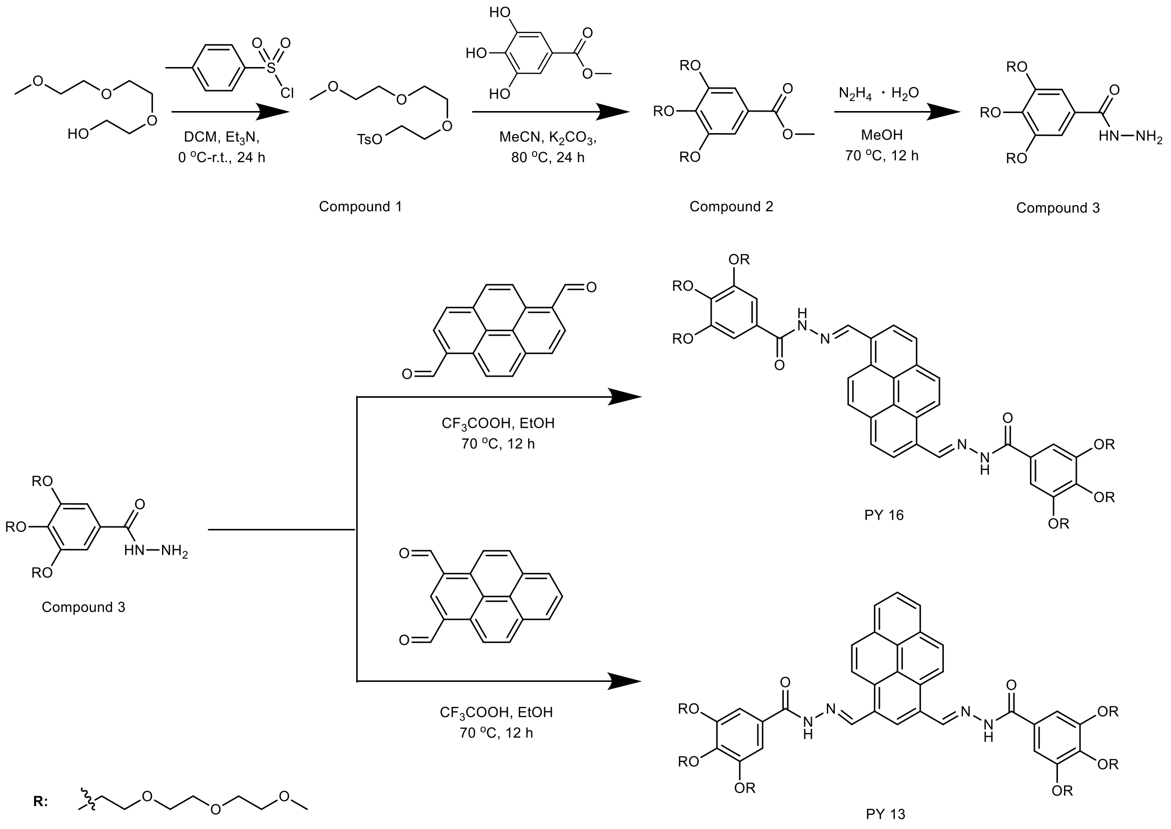


**Supplementary Figure 1.** Synthesis of compounds **PY16** and **PY13**.

Compounds **1-3** were synthesized according to previous reports. [1-3]

Compound **1** synthesis: To a 1 L flask was added triethylene glycol monomethyl ether (19.2 mL, 120 mmol), Triethylamine (20.8 mL, 150 mmol), dichloromethane (150 mL). p-Toluenesulfonyl chloride (19.1 g, 100 mmol) in dichloromethane (300 mL) was slowly dropped into the mixture at 0 ℃ with stirring, then returned to room temperature and stired for about 24 h. The solvent was evaporated by a rotary evaporator to afford a concentrated suspension (about 100 mL) and then filtered. The filtrate was washed with saturated brine (3x30 mL). The organic phase was dried with anhydrous sodium sulfate and filtered. The filtrate was evaporated to give compound **1** (22.0 g, 69%) as viscous liquid. It was taken to the next step without any further

purification. $^1$H NMR (400 MHz, Chloroform-d, δ): 7.83-7.76 (m, 2H), 7.34 (d, J = 8.1 Hz, 2H), 4.19-4.13 (m, 2H), 3.71-3.66 (m, 2H), 3.64-3.58 (m, 6H), 3.56-3.50 (m, 2H), 3.37 (s, 3H), 2.44 (s, 3H).

Compound **2** synthesis: To a 1 L flask was added Compound **1** (25.2 g, 79.2 mmol), methyl gallate (2.8 g, 15.2 mmol), potassium carbonate (7.4 g, 53.2 mmol), and acetonitrile (400 mL). The mixture was refluxed under nitrogen protection for about 24 hours. Then the reaction solution was filtered and washed with dichloromethane. The filtrate was rotary evaporated to afford crude product and then purified by silica gel column chromatography (dichloromethane/MeOH = 50:1 v/v) to give compound **2** (5.6 g, 59%) as oil. $^1$H NMR (400 MHz, Chloroform-d, δ): 7.20 (s, 2H), 4.16-4.08 (m, 6H), 3.90-3.78 (m, 7H), 3.71 (t, J = 5.0 Hz, 2H), 3.66-3.50 (m, 24H), 3.30 (s, 9H).

Compound **3** synthesis: Hydrazine monohydrate (2 mL, 40.14 mmol) was added to a solution of compound **2** (800 mg, 1.284 mmol) in MeOH (20 mL). The reaction mixture was heated to reflux for 12 hours under inert atmosphere. Then reaction was stopped and cooled to room temperature and methanol was evaporated. The product was extracted with dichloromethane (100 mL) and washed with brine solution (3x20 mL). The organic layer was dried over anhydrous $Na_2SO_4$ and solvent was evaporated to to afford crude product. Then the crude product was further purified by silica gel column chromatography (dichloromethane/MeOH = 15:1, v/v) to provide compound **3** (576 mg, 72% yield) as brown oil. $^1$H NMR (400 MHz, DMSO-$d_6$, δ): 9.68 (s, 2H), 7.16 (s, 2H), 4.14 (t, J = 4.0 Hz, 4H), 4.06 (t, J = 4.0 Hz, 2H), 3.76 (t, J = 4.0 Hz, 4H), 3.67 (t, J = 4.0 Hz, 2H), 3.62-3.49 (m, 18H), 3.43-3.41 (m, 6H), 3.24 (s, 9H).

Compound **PY16** synthesis: Compound **3** (482.2 mg, 0.775 mmol) was added to a suspension of 1,6-diformylpyrene (100 mg, 0.388 mmol) in EtOH (150 mL). Trifluoroacetate (0.442 mg, 0.00388 mmol) diluted in EtOH (5 mL) was added dropwise to the suspension. The reaction mixture was heated to reflux for 12 h and evaporated to afford crude product (brown oil) after

cooling to room temperature. Then the crude product was further purified by silica gel column chromatography (dichloromethane/MeOH = 15:1, v/v) to provide **PY16** (456 mg, 80% yield) as yellow oil; $^1$H NMR (400 MHz, DMSO-d$_6$, δ): 12.16 (s, 2H), 9.66 (s, 2H), 8.89 (d, J = 9.3 Hz, 2H), 8.63 (d, J = 8.0 Hz, 2H), 8.43 (d, J = 8.2 Hz, 2H), 8.39 (d, J = 9.3 Hz, 2H), 7.39 (s, 4H), 4.26-4,24 (m, 8H), 4.13 (t, J = 6.6 Hz, 4H), 3.81 (s, 8H), 3.71 (t, J = 7.2 Hz, 4H), 3.65-3.62 (m, 8H), 3.61-3.58 (m, 4H), 3.56-3.55 (m, 8H), 3.53-3.50 (m, 16H), 3.44-3.41 (m, 12H), 3.24 (s, 6H), 3.23 (s, 12H); $^{13}$C NMR (151 MHz, DMSO-*d*$_6$, δ): 162.60, 152.10, 146.26, 140.77, 131.48, 129.71, 128.94, 128.83, 128.23, 128.02, 125.99, 125.49, 124.32, 123.55, 107.02, 72.03, 71.34, 70.06, 69.96, 69.93, 69.84, 69.68, 69.05, 68.71, 58.10; HRMS (ESI) (m/z): $[M+Na]^+$ calcd for $C_{74}H_{106}N_4O_{26}Na^+$, 1489.6988; found, 1489.6986.

Compound **PY13** synthesis: Compound **3** (482.2 mg, 0.775 mmol) was added to a suspension of 1,3-diformylpyrene (100 mg, 0.388 mmol) in EtOH (150 mL). Trifluoroacetate (0.442 mg, 0.00388 mmol) diluted in EtOH (5 mL) was added dropwise to the suspension. The reaction mixture was heated to reflux for 12 h and evaporated to afford crude product (brown oil) after cooling to room temperature. Then the crude product was further purified by silica gel column chromatography (dichloromethane/MeOH = 15:1, v/v) to provide **PY13** (416 mg, 73% yield) as yellow oil; $^1$H NMR (400 MHz, DMSO-*d*$_6$, δ): 11.99 (s, 2H), 9.58 (s, 2H), 9.14 (s, 1H), 8.82 (d, J = 8.0 Hz, 2H), 8.47 (s, 2H), 8.45 (s, 2H), 8.19 (t, J = 7.6 Hz, 1H), 7.34 (s, 4H), 4.26-4.24 (m, 8H), 4.14 (t, J = 4.4 Hz, 4H), 3.83-3.80 (m, 8H), 3.71 (t, J = 4.8 Hz, 4H), 3.66-3.62 (m, 8H), 3.61-3.58 (m, 4H), 3.57-3.56 (m, 8H), 3.54-3.50 (m, 16H), 3.45-3.41 (m, 12H), 3.24 (s, 6H), 3.23 (s, 12H); $^{13}$C NMR (101 MHz, DMSO-*d*$_6$, δ): 162.63, 152.11, 145.91, 140.73, 130.49, 129.70, 128.29, 127.11, 126.90, 124.84, 123.94, 122.41, 106.94, 72.03, 71.34, 70.06, 69.92, 69.83, 69.68, 69.04, 68.68, 58.10; HRMS (ESI) (m/z): $[M+Na]^+$ calcd for $C_{74}H_{106}N_4O_{26}Na^+$, 1489.6988; found, 1489.6996.

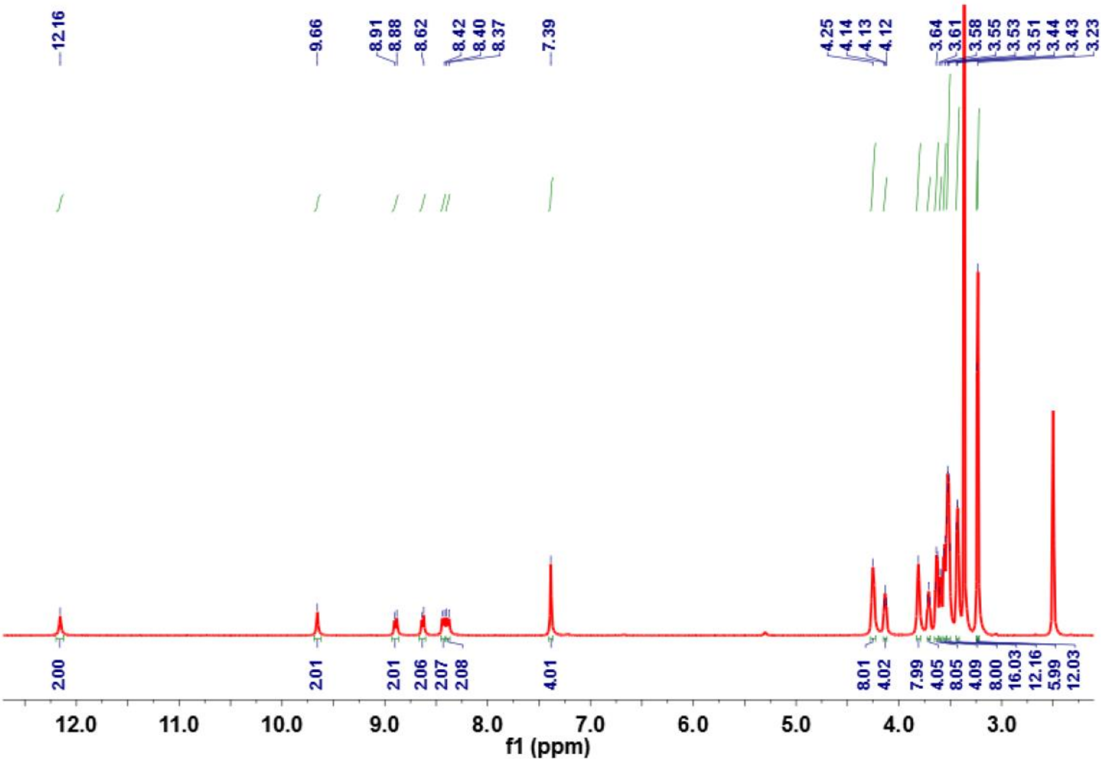


**Supplementary Figure 2.** $^{1}H$ NMR spectrum of compound **PY16**.

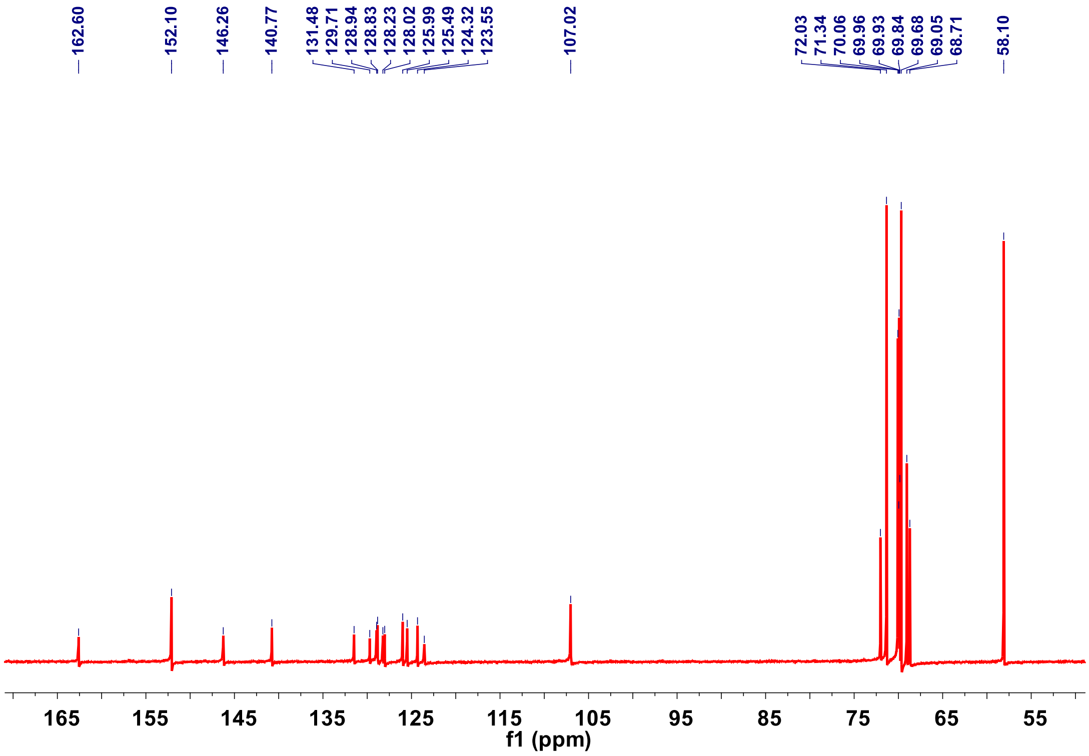


**Supplementary Figure 3.** $^{13}C$ NMR spectrum of compound **PY16**.

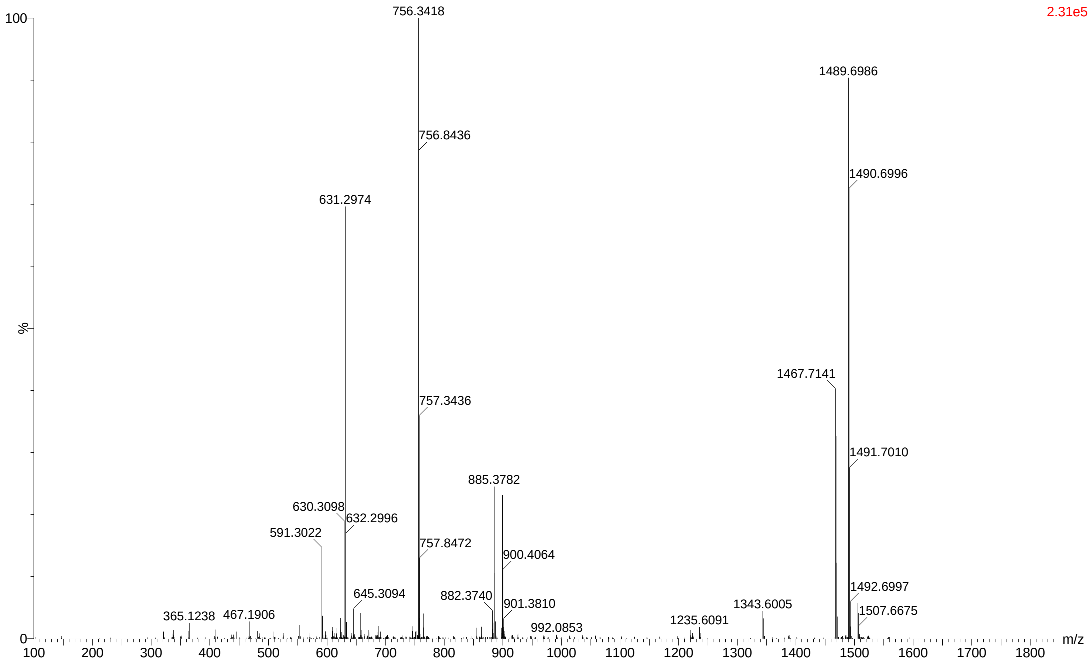


**Supplementary Figure 4.** Mass spectrum of compound **PY16**.

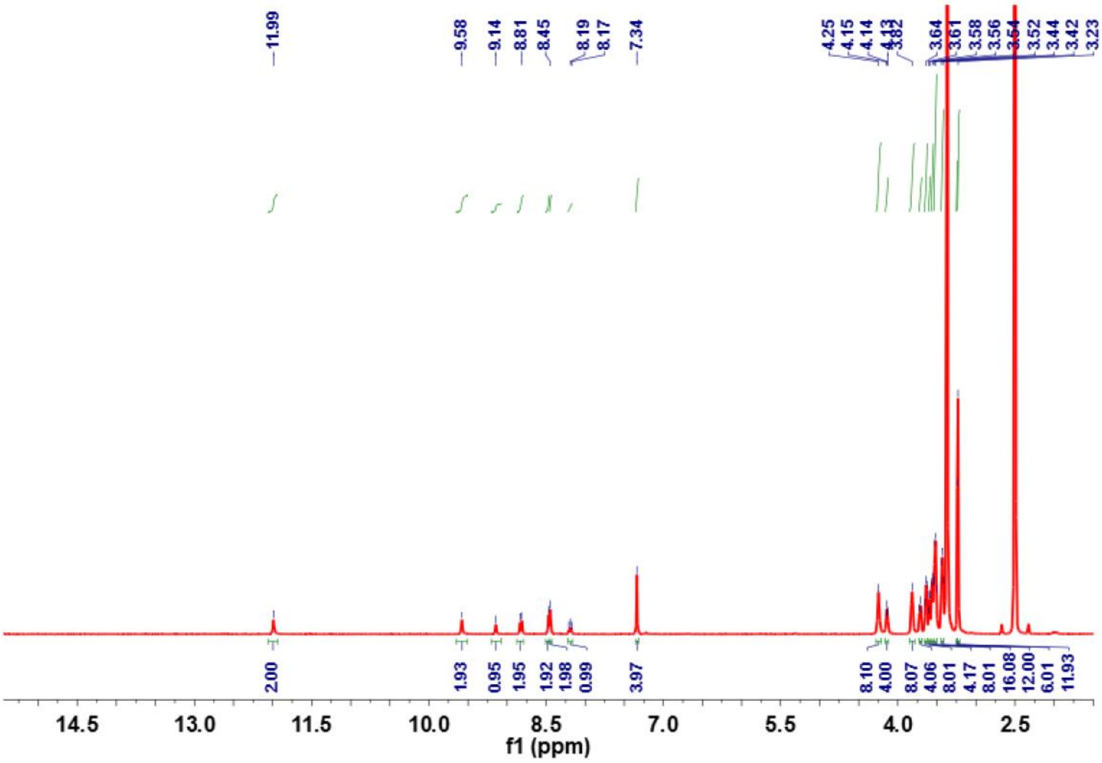


**Supplementary Figure 5.** $^{1}$H NMR spectrum of compound **PY13**.

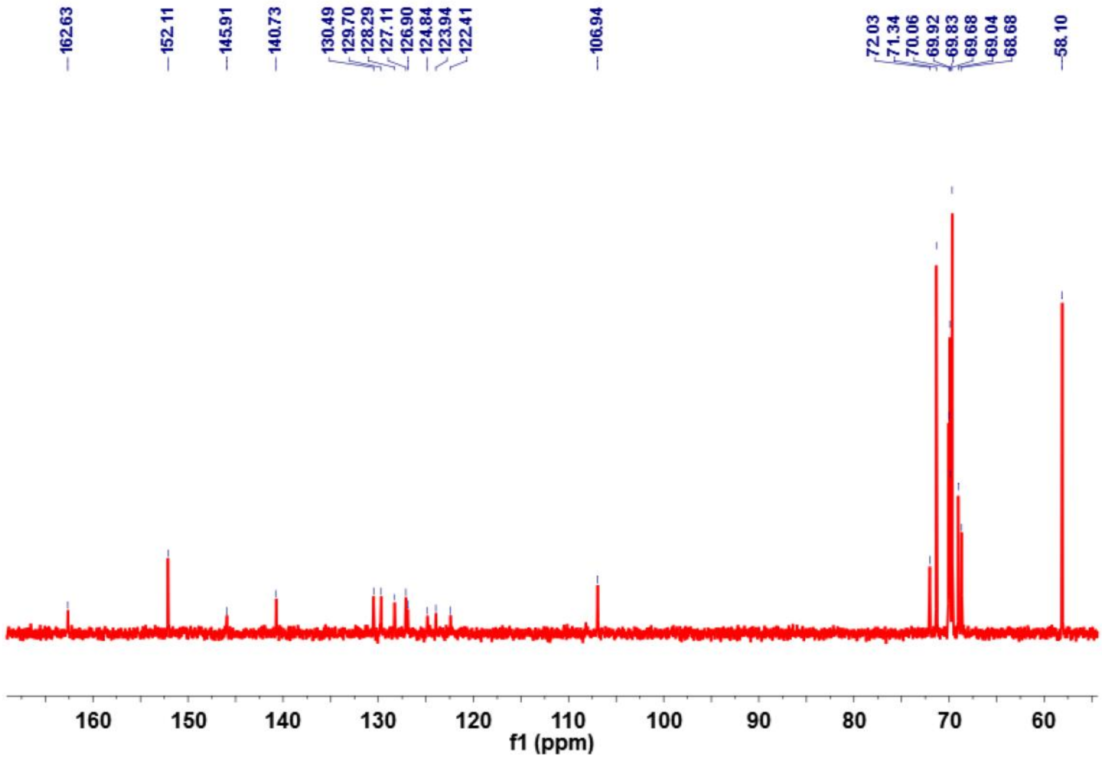


**Supplementary Figure 6.** $^{13}$C NMR spectrum of compound **PY13**.

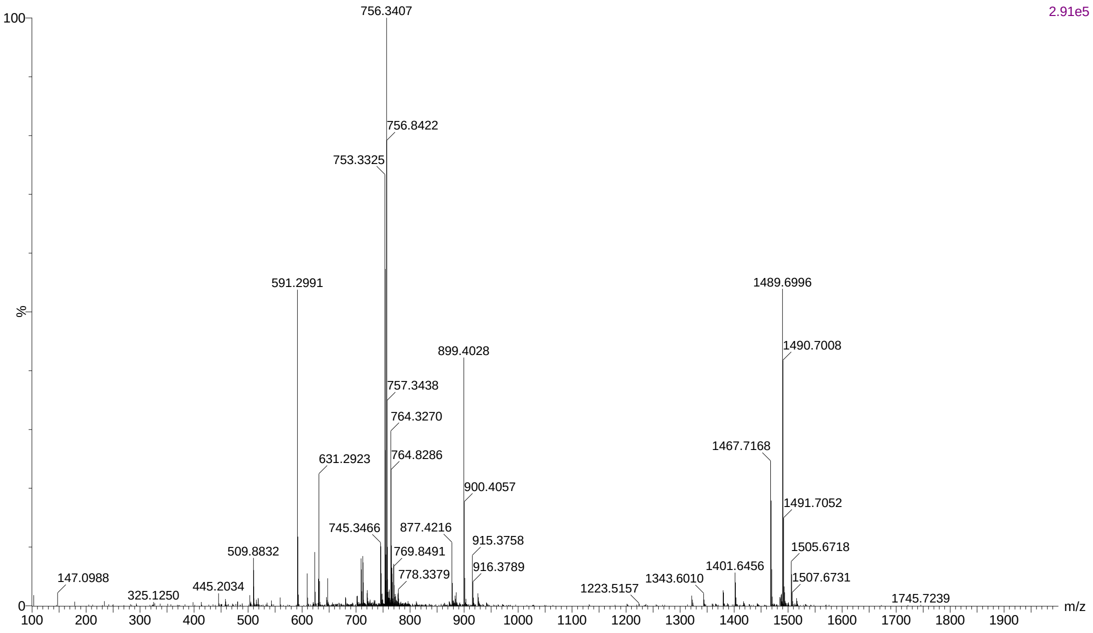


**Supplementary Figure 7.** Mass spectrum of compound **PY13**.

# 2. Supplementary Figures

## 2.1 Amphiphilic self-assembly

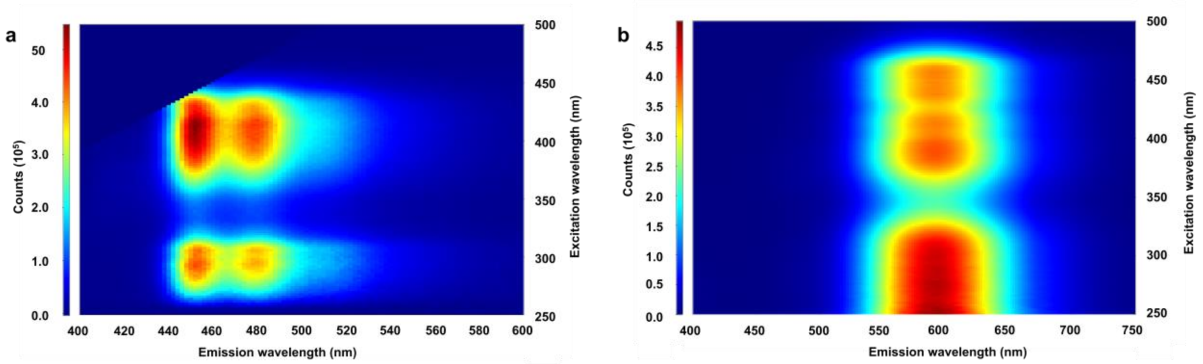


**Supplementary Figure 8.** Excitation fluorescence mapping of compound **PY16** (a) DMSO solution, (b) aqueous solution (25 °C, 20 μM).

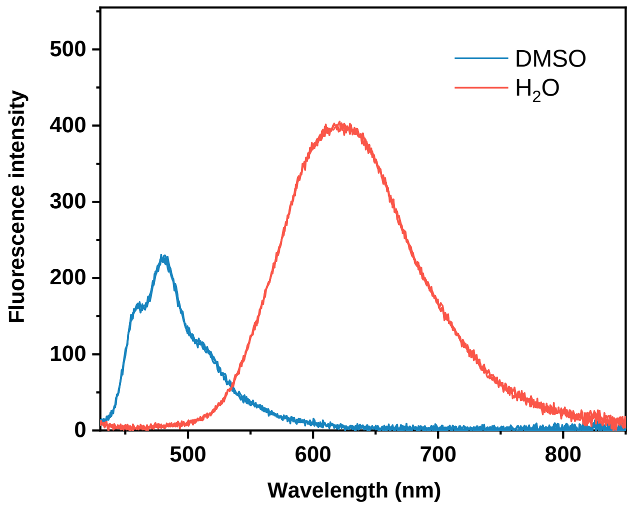


**Supplementary Figure 9.** The absolute fluorescence quantum yield of compound **PY16** in DMSO and aqueous solutions (0.1 mM, $\lambda_{ex}$ = 400 nm). The measurement was repeated for three times and the average absolute fluorescence quantum yield was 8.0% (DMSO) and 39.5% ($H_2O$), respectively.

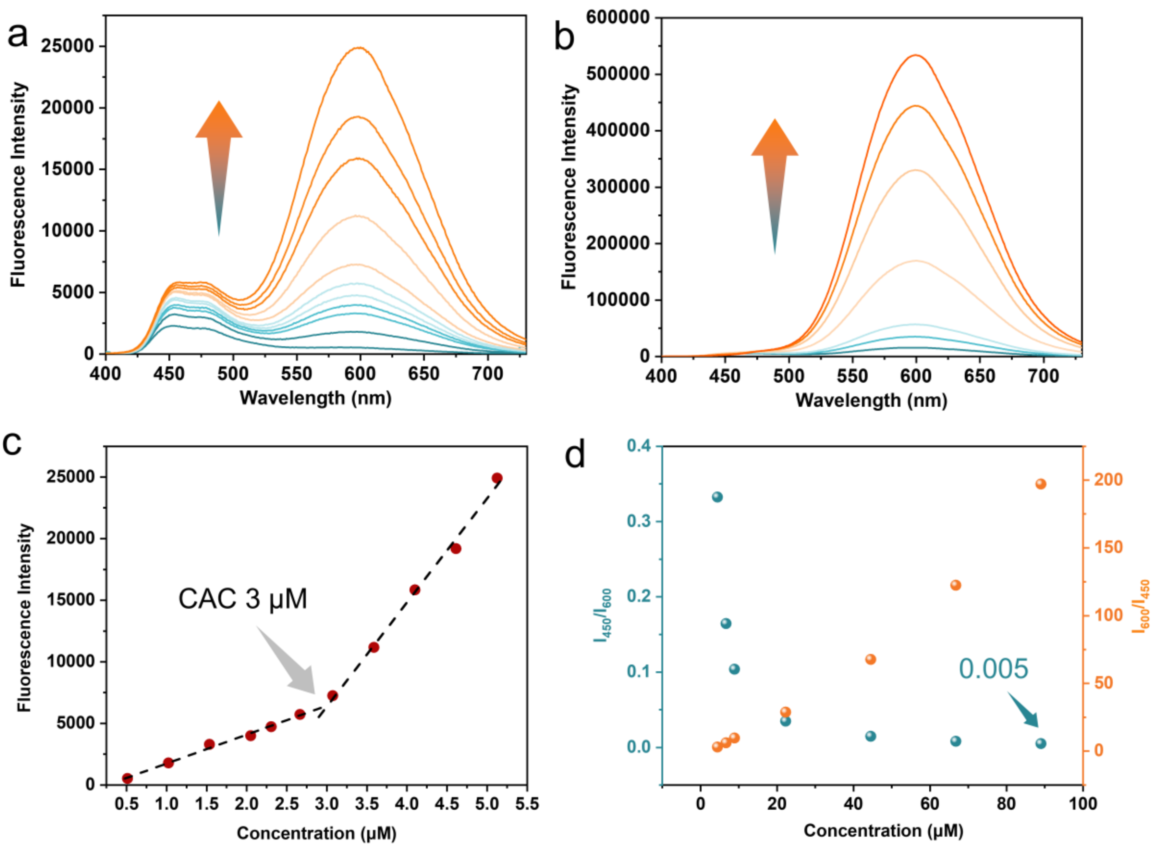


**Supplementary Figure 10.** Concentration-dependent fluorescence spectra of compound **PY16** in aqueous solutions: (a) from 0.45 to 5.5 µM; (b) from 4.5 to 89 µM, $\lambda_{ex}$ = 380 nm. (c) The plot of the fluorescence intensity at 600 nm versus the concentration, revealing the critical assembly concentration as 3 µM. (d) The plot of the fluorescence intensity ratio versus the monomeric PY16 concentration.

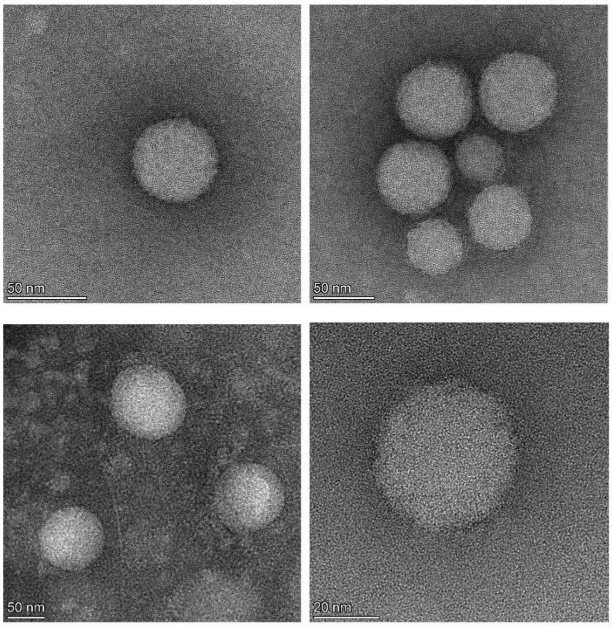


**Supplementary Figure 11.** TEM images of compound **PY16** in an aqueous solution (0.1 mM).

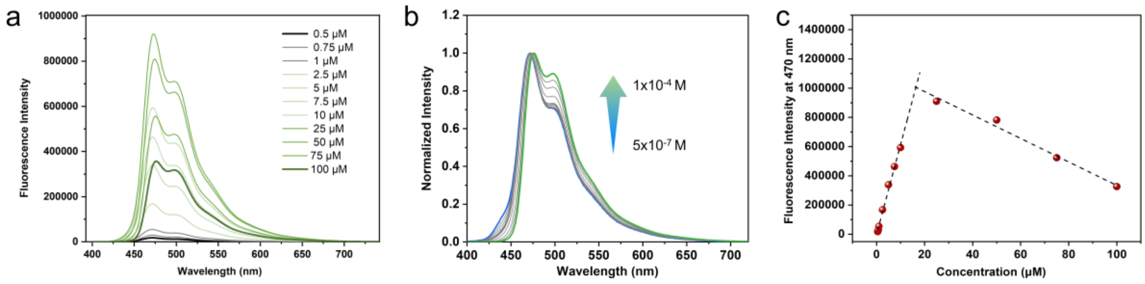


**Supplementary Figure 12.** Concentration-dependent (0.5-100 μM) (a) fluorescence spectra and (b) normalized fluorescence spectra of compound **PY13** in aqueous solutions. (c) The plot of the fluorescence intensity at 470 nm versus **PY13** concentration. The decrease of intensity at high concentrations (above 18 μM) may be attributed to the inner filter effect.

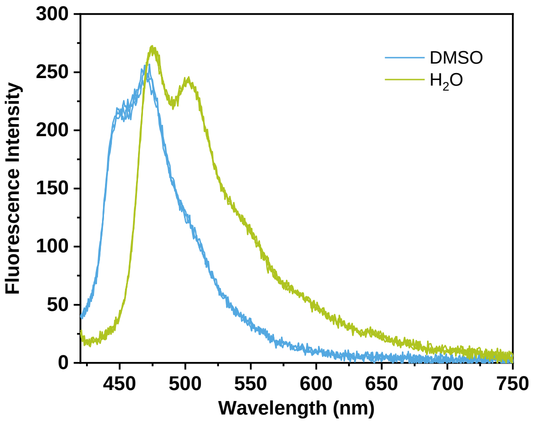


**Supplementary Figure 13.** The absolute fluorescence quantum yield of compound **PY13** in DMSO and aqueous solutions (0.1 mM, $\lambda_{ex}$ = 380 nm). The measurement was repeated for three times and the average absolute fluorescence quantum yield was 6.7% (DMSO) and 13.2% ($H_2O$), respectively.

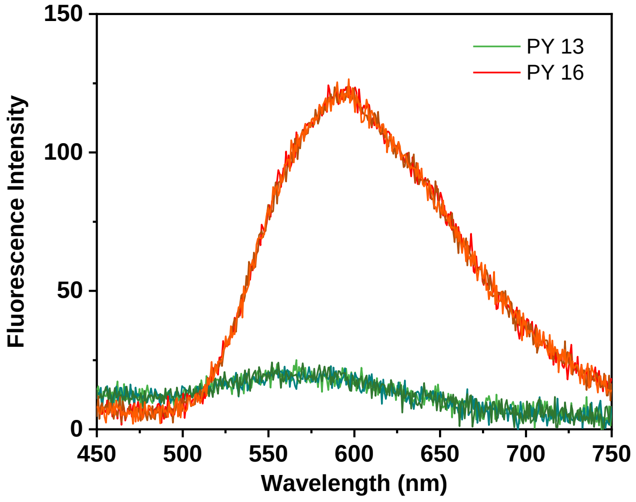

**Supplementary Figure 14.** The absolute solid fluorescence quantum yield of compounds **PY16** and **PY13**. The film was prepared by dropping compounds' aqueous solution into a quartz flake and then evaporated. Each sample was measured three times and the average absolute solid fluorescence quantum yield was 15.4% (**PY16**) and 6.6% (**PY13**), respectively.

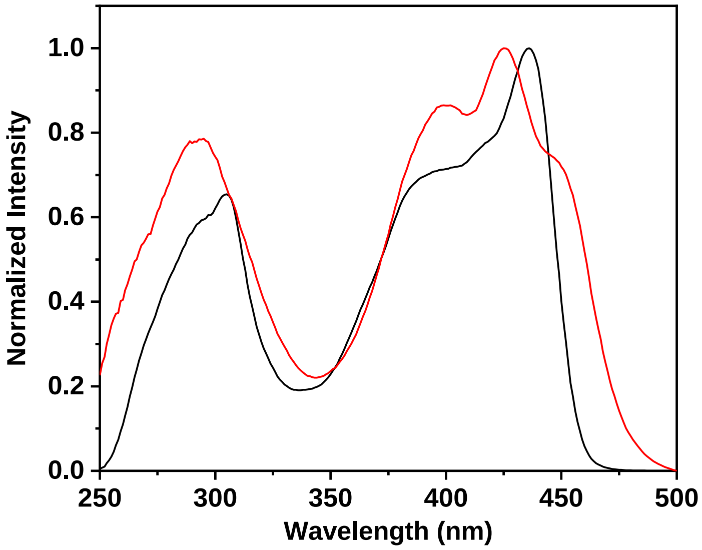


**Supplementary Figure 15.** Excitation spectra of compound **PY16** (black line: DMSO, $\lambda_{em}$ = 450 nm; red line: $H_2O$, $\lambda_{em}$ = 600 nm, 20 μM).

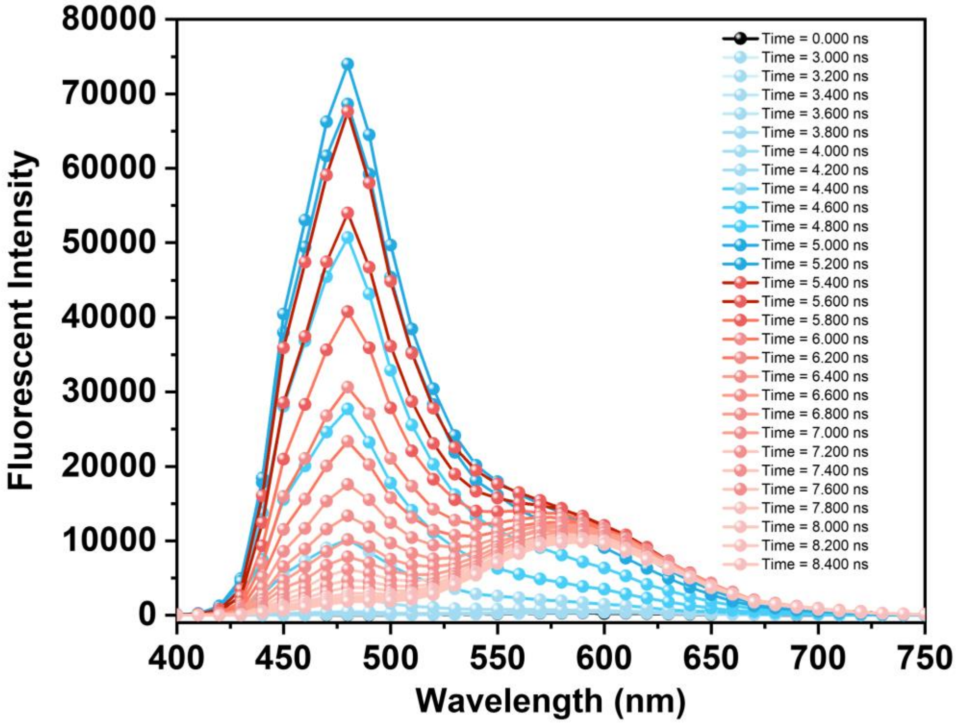


**Supplementary Figure 16.** Time-resolved fluorescence spectra of compound **PY16** in DMSO/$H_2O$ mixture with 90% water fraction (20 μM).

## 2.2 Information encryption materials

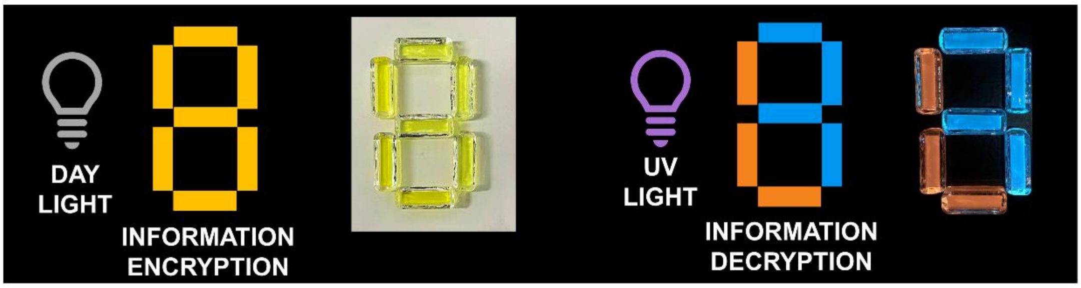


**Supplementary Figure 17.** The photographs of **PY16** solution in natural light and UV light.

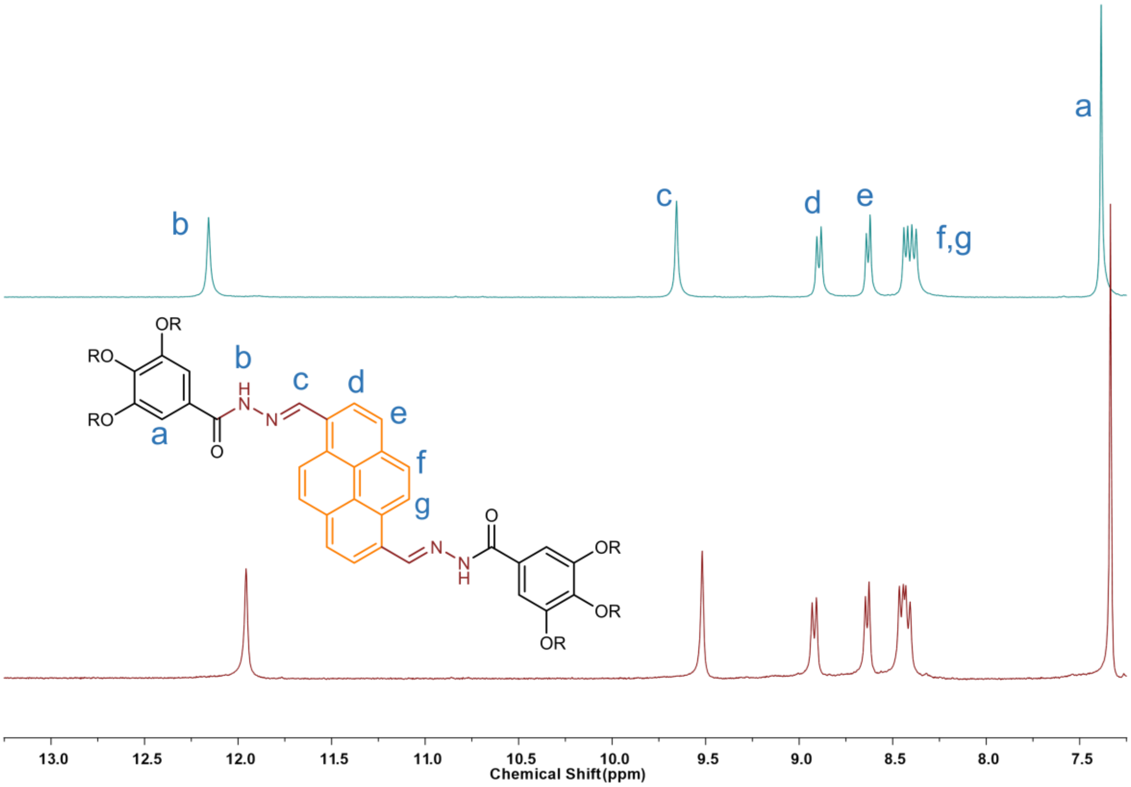


**Supplementary Figure 18.** $^1$H NMR spectra of compound **PY16** before (green line) and after (red line) staying in water for three days. Compound **PY16**'s aqueous solution (0.1 mM) was stayed in the dark for 3 days and then freeze-dried, redissolved in DMSO-$d_6$. The proton signal of the pyrene and dynamic acylhydrazone unit showed similar chemical shift and split to the previous one, suggesting that **PY16** was stable in aqueous solution.

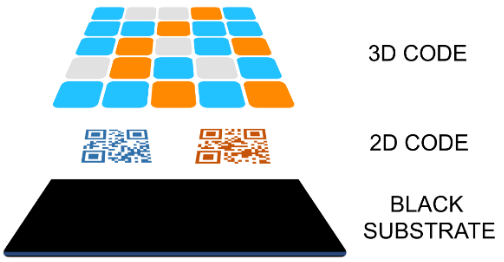


**Supplementary Figure 19.** A schematic illustration for the formation of “sandwich” structure.

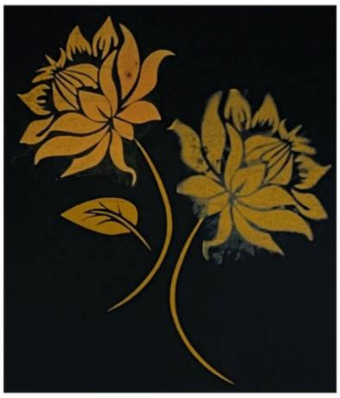

**Supplementary Figure 20.** Fluorescent pattern “rose” after solvent evaporation.

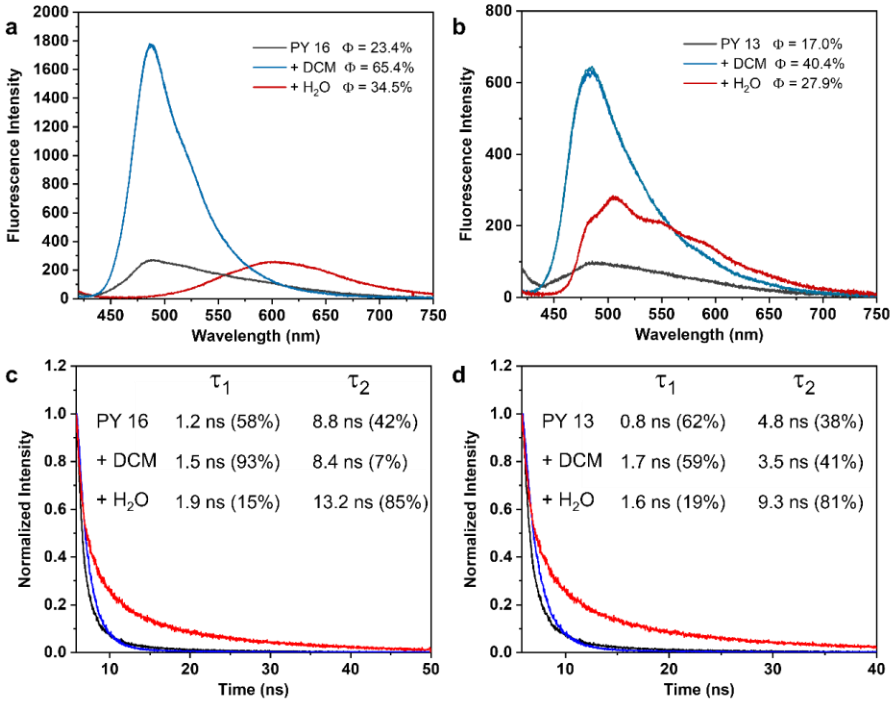


**Supplementary Figure 21.** The absolute fluorescence quantum yield (Φ) of compound (a) **PY16** and (b) **PY13** absorbed on silica gel, before (black line) and after solvent treatment (blue line: dichloromethane, red

line: water). The measurements were repeated for three times and the average absolute fluorescence quantum yield was shown in the figure. The fluorescence lifetimes (τ) of compound (c) **PY16** and (d) **PY13** absorbed on silica gel, before (black line) and after solvent treatment (blue line: dichloromethane, red line: water). Both of the compounds exhibited a longer lifetime after water treatment, suggesting the occurrence of assembly.

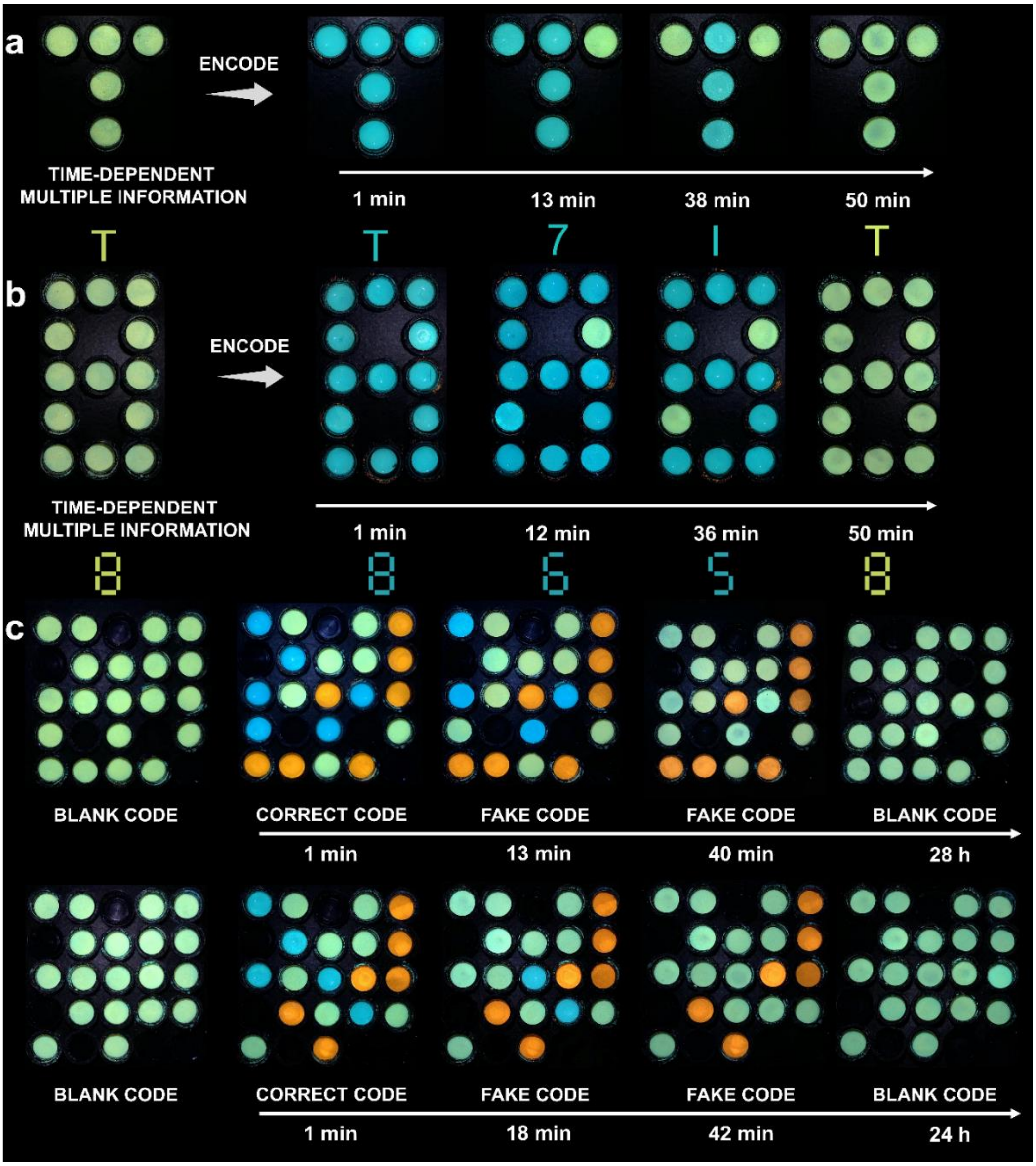


**Supplementary Figure 22.** Photographs of encrypted letter (a) "T" and (b) "8" before and after DCM treatment, displaying time-dependent multiple information in which the correct information can be identified only at a specified time. (c) Photographs of the time-dependent 3D code (4D code) with varied information, correct/fake/black code, on a time scale of 1 minute to 28 hours.

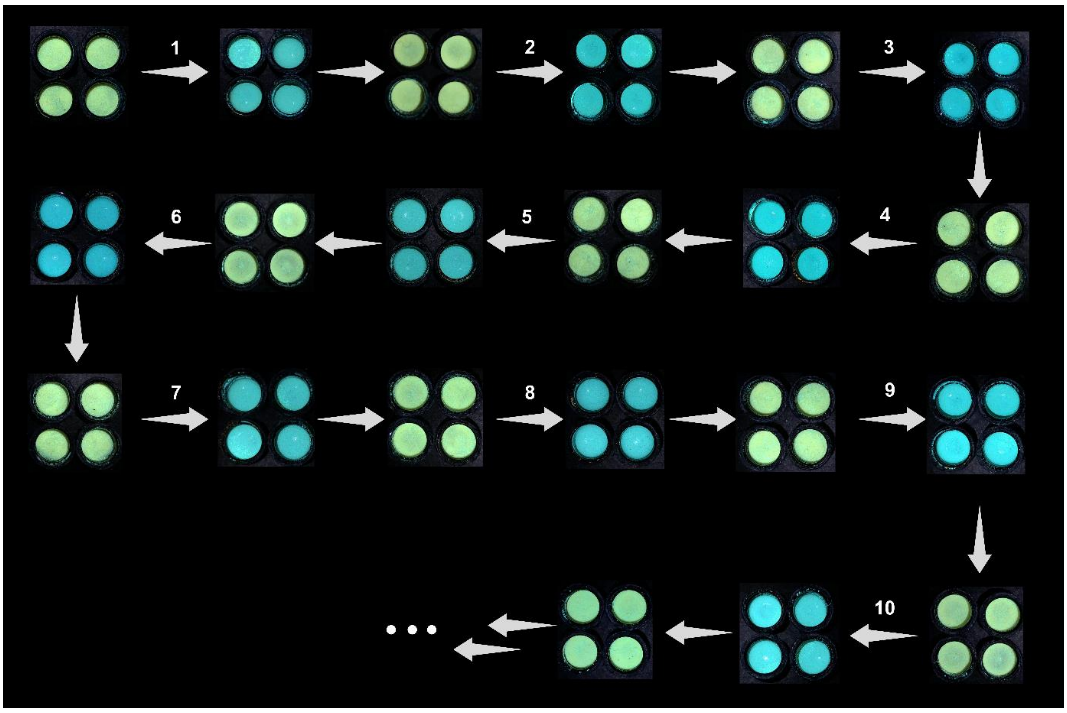


**Supplementary Figure 23.** Illustrations of 10 repeating cycles of dynamic fluorescence material.

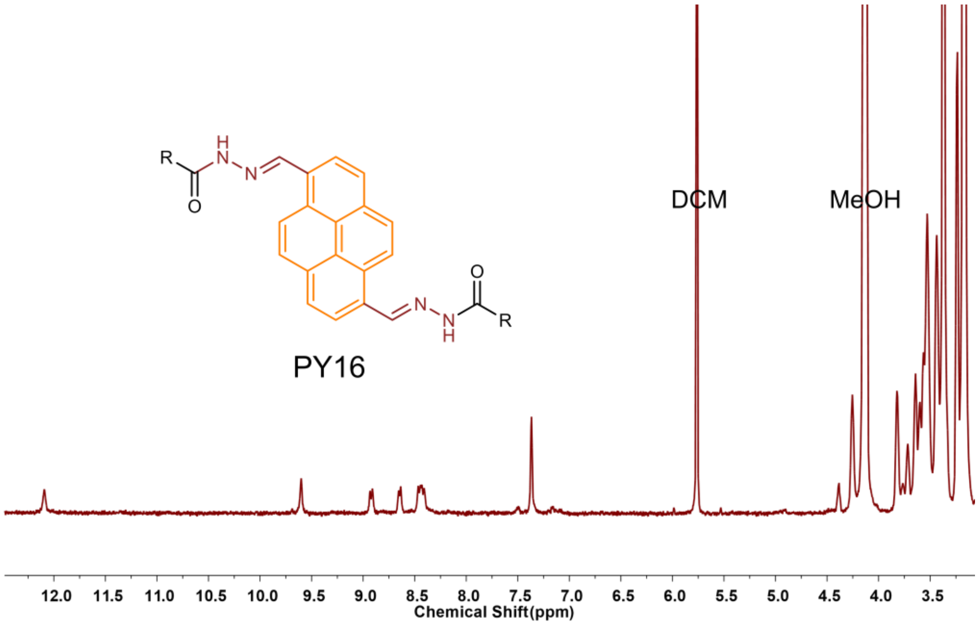


**Supplementary Figure 24.** $^{1}H$ NMR spectrum of compound **PY16** after performing ten written-erased cycles. Similar proton signals were observed comparing to the compound without absorbing on silica gel (Supplementary Fig. 2), indicating good stability of this compound absorbed on silica gel.

## 3. Supplementary References


1. Wang, Q., Zhang, Q., Zhang, Q.-W., Li, X., Zhao, C.-X., Xu, T.-Y., Qu, D.-H. & Tian H. Color-tunable single-fluorophore supramolecular system with assembly-encoded emission. *Nat. Commun.* **11**, 158 (2020).

2. Wang, F., Wang, W.-G., Wang, X.-J., Wang, H.-Y., Tung, C.-H. & Wu, L.-Z. A highly efficient photocatalytic system for hydrogen production by a robust hydrogenase mimic in an aqueous solution. *Angew. Chem. Int. Ed.* **50**, 3193-3197 (2011).

3. Molla, M. R. & Ghosh, S. Hydrogen-bonding-mediated vesicular assembly of functionalized naphthalene-diimide-based bolaamphiphile and guest-induced gelation in water. *Chem. Eur. J.* **18,** 9860-9869 (2012).
