# Supplementary material for: A dynamic assembly-induced emissive system for advanced information encryption with time-dependent security
Source: Nat Commun. 2022 Jul 20;13:4185. doi: 10.1038/s41467-022-31978-x (PMC9300691; doi:10.1038/s41467-022-31978-x)
Supplement: Supplementary file 2 — Description of Additional Supplementary Files [file 41467_2022_31978_MOESM2_ESM.pdf]

Supplementary Movie 1: The recognition of code A through a smartphone, resulting in information “A”.

Supplementary Movie 2: The recognition of code B through a smartphone, resulting in information “2021”.

Supplementary Movie 3: The recognition of code C through a smartphone, resulting in information “KLAM”.

Supplementary Movie 4: The recognition of red 2D code, resulting in information “A”.

Supplementary Movie 5: The recognition of blue 2D code, resulting in information “B”.

Supplementary Movie 6: The recognition of 3D code under UV light, resulting in information “FLUORESCENCE”.

Supplementary Movie 7: The recognition of 3D code under UV light, resulting in information “color”.

Supplementary Movie 8: The recognition of red 2D code under UV light, resulting in information “A”.

Supplementary Movie 9: The fluorescence colour change of the printing pattern with time, owing to solvent evaporation. Movie speed up: 26×.

Supplementary Movie 10: The silica gel powder containing compound PY16 was loaded in a black container. The addition of dichloromethane resulted in blue fluorescence that automatically reverted back to yellow with time. Movie speed up: 250×.
